# Proteomic Insights into Phycobilisome Degradation, A Selective and Tightly Controlled Process in The Fast-Growing Cyanobacterium *Synechococcus elongatus* UTEX 2973

**DOI:** 10.3390/biom9080374

**Published:** 2019-08-16

**Authors:** Aparna Nagarajan, Mowei Zhou, Amelia Y. Nguyen, Michelle Liberton, Komal Kedia, Tujin Shi, Paul Piehowski, Anil Shukla, Thomas L. Fillmore, Carrie Nicora, Richard D. Smith, David W. Koppenaal, Jon M. Jacobs, Himadri B. Pakrasi

**Affiliations:** 1 Department of Biology, Washington University, St. Louis, MO 63130, USA; 2 Environmental and Molecular Sciences Laboratory, Pacific Northwest National Laboratory, Richland, WA 99354, USA; 3 Biological Sciences Division, Pacific Northwest National Laboratory, Richland, WA 99354, USA

**Keywords:** cyanobacteria, phycobilisomes, light harvesting complexes, protein degradation, nitrogen starvation, proteomics, photosynthesis

## Abstract

Phycobilisomes (PBSs) are large (3–5 megadalton) pigment-protein complexes in cyanobacteria that associate with thylakoid membranes and harvest light primarily for photosystem II. PBSs consist of highly ordered assemblies of pigmented phycobiliproteins (PBPs) and linker proteins that can account for up to half of the soluble protein in cells. Cyanobacteria adjust to changing environmental conditions by modulating PBS size and number. In response to nutrient depletion such as nitrogen (N) deprivation, PBSs are degraded in an extensive, tightly controlled, and reversible process. In *Synechococcus elongatus* UTEX 2973, a fast-growing cyanobacterium with a doubling time of two hours, the process of PBS degradation is very rapid, with 80% of PBSs per cell degraded in six hours under optimal light and CO_2_ conditions. Proteomic analysis during PBS degradation and re-synthesis revealed multiple proteoforms of PBPs with partially degraded phycocyanobilin (PCB) pigments. NblA, a small proteolysis adaptor essential for PBS degradation, was characterized and validated with targeted mass spectrometry. NblA levels rose from essentially 0 to 25,000 copies per cell within 30 min of N depletion, and correlated with the rate of decrease in phycocyanin (PC). Implications of this correlation on the overall mechanism of PBS degradation during N deprivation are discussed.

## 1. Introduction

Cyanobacteria are photosynthetic microbes that can convert sunlight into chemical energy using CO_2_ as a carbon source. In cyanobacteria, the protein complexes of the photosynthetic electron transport chain are located in the internal thylakoid membrane system. Large extrinsic phycobilisome (PBS) antenna complexes associate with the cytoplasmic side of the thylakoid membranes and harvest light energy for the photosynthetic apparatus [1,2]. Considerable attention has been placed on cyanobacteria based on their potential to produce biofuels and biochemicals in a carbon-neutral manner. Unfortunately, most cyanobacteria to date have shown slow rates of growth and biomass accumulation compared to heterotrophic organisms such as yeast, precluding their use in industrial settings. However, recent work with the cyanobacterium *Synechococcus elongatus* UTEX 2973 (hereafter referred to as UTEX 2973) has demonstrated that this strain possesses a robust growth phenotype with a doubling time of 1.5 h and greater biomass accumulation in comparison to other more commonly used model cyanobacteria [3]. Interestingly, the faster growth rate in UTEX 2973 is attributed to adjustments in the photosystem and light harvesting components [4]. Given that many details of PBS modulation and regulation are not well-characterized in cyanobacteria, it is important to understand how cyanobacteria respond to changing environmental conditions in order to optimize the use of these organisms as bioproduction platforms.

PBS are large membrane extrinsic antenna systems that are composed of different pigment-proteins called phycobiliproteins (PBPs) and non-pigmented linker proteins, all serving as building blocks that assemble to form massive complexes [5]. The three major types of cyanobacterial PBPs are phycocyanin (PC), allophycocyanin (APC), and phycoerythrin (PE) [6]. These PBPs are assembled into the hemidiscoidal or hemielliptical structure of the PBS, with APC forming the core, and PC and PE (if present) forming rods that radiate from the core. The composition and organization of PBS vary between different cyanobacteria. PBSs in UTEX 2973 are likely similar to the closely related model strain *Synechococcus elongatus* PCC 7942, having a dicylindrical core and a set of six cylindrical rods that radiate from the core [7]. PBS content in cyanobacteria can be estimated based on the spectroscopic determination of PC since these PBPs dominate PBS complexes [8,9]. Other types of cyanobacterial PBSs are well characterized, including the tricylindrical core of the PBSs in *Synechocystis* sp. PCC 6803 [10] and the pentacylindrical core and eight peripheral rods of the PBSs in *Anabaena* sp. PCC 7120 [11]. PBSs can account for a significant percentage of the soluble proteins in cyanobacteria (i.e., up to 50%) [7], thus serving as a large potential nutrient source in cyanobacterial cells.

The ordered assembly of PBS typically begins with the nucleation of the pigmented α-phycocyanin (CpcA) and β-phycocyanin (CpcB) subunits to form a PC monomer that serves as the building block for the PBS complex [5]. Six PC monomers form a PC hexamer, and usually three PC hexamers are linked together with the help of colorless linker proteins such as CpcD, the most distal linker protein, to form a PC rod [7]. Generally, six peripheral PC rods radiate from the allophycocyanin (APC) core, which is made up of α-APC (ApcA) and β-APC (ApcB) subunits. ApcD and ApcF are α- and β-APC like proteins, respectively, that make up one of the core trimers and are involved in energy transfer to the photosystems along with ApcE [12]. More than a hundred monomers of α- and β-PC covalently associate with a total of more than three hundred phycocyanobilins (PCB) to make up one PBS complex, highlighting the enormous amount of PBPs that must come together to make these massive complexes [6].

Regulation of light harvesting in cyanobacteria is critical for cell survival during changing environmental conditions. PBSs play an integral role in protecting cyanobacteria from environmental stresses by serving as large nutrient reserves due to the massive number of PBPs required to create them. The dynamic remodeling of PBSs provides a mechanism for cyanobacteria to fine-tune their responses to fluctuating nutrient and light availability [7,13]. Low-level photosynthesis and loss of pigments are crucial for cell survival during nitrogen, sulfur, or phosphorous depletion [14]. The degradation of PBSs provides amino acids that are essential for the maintenance of central metabolic activities and as a form of photoprotection [5,6]. As cellular metabolism slows during nutrient depletion, the amount of light energy harvested needs to decrease to prevent reactive oxygen species (ROS) from being created. Consequently, PBS degradation plays an important function in photoprotection, cell maintenance, growth, and development [15].

The phenomenon of PBS degradation was observed more than four decades ago, and is a process in which cyanobacteria undergo bleaching due to PBS degradation during nitrogen, sulfur, or phosphorus deprivation [16,17,18]. Previous studies of PBS degradation have been in model organisms where this process occurs over a period of 24–48 h during nutrient deprivation [19,20]. PBS degradation is a reversible process and PBSs are resynthesized immediately upon exogenous addition of nutrients. PBS degradation is a rapid, specific, and extensive process given the large amount of protein complexes involved in the process. Proteins involved in PBS degradation include, but are not limited to, NblA, NblR, NblS, NblB, RpaB, and NtcA [15,21,22,23,24]. One of the central proteins is NblA, named because of the nonbleaching phenotype in mutant strains lacking Non-bleaching A (NblA) [25]. NblA is a small 7 kilodalton (KDa) adaptor protein that facilitates the interaction of a Clp protease with PBPs, which triggers PBS degradation [26]. However, despite the large scale of this degradation event, to date the stoichiometric relationship of the NblA protein to PBS remains unknown. In order to understand the mechanism of PBS degradation, it is useful to know the number of NblA proteins present in cells during nitrogen starvation and how NblA responds to nitrogen re-addition.

Mass spectrometry (MS) based proteomics has been widely applied to identify and quantify large numbers of proteins in complex biological samples [27,28,29]. By comparing the molecular features between samples at different states, proteins that are changing significantly can be readily identified, offering enormous insights into the underlying mechanism of biological pathways. Typically, proteins extracted from biological samples are first digested with specific proteases such as trypsin to generate predictable peptides of a few KDa in mass that are then identified using liquid chromatography (LC) coupled to MS. The resulting peptides can be used as proxy to infer the presence or amount of proteins in the sample. This “bottom-up” proteomics approach is very sensitive and powerful, and widely applied for proteomics studies.

Recently, advanced MS technology has increasingly allowed for bypassing the proteolysis step to enable “top-down” proteomics analysis of intact proteins [30,31], and even noncovalent native protein complexes up to megadalton (MDa) mass range (i.e., native MS or nondenaturing MS) [32]. These new approaches provide opportunities to characterize not only the presence of proteins but also their coexisting variations or modifications (i.e., proteoforms), including binding partners. Although technical challenges still exist, top-down proteomics provides tremendous benefits, for example, by revealing the endogenous proteoforms bearing multiple modifications or truncations simultaneously [33]. Label-free top-down quantitation has been shown to effectively capture changes of intact proteoforms that are more tightly related to phenotypes than peptides in bottom up quantitation [34,35]. Light harvesting pigments are known to covalently bind PBPs through the sulfur ester on cysteines [5]. Characterization of these covalent interactions specifically under nutrient starvation will provide additional information towards their possible mechanism of degradation. Currently, little detail is known at the protein level about PBS degradation, including the mechanism and process of degradation of the individual PBS protein components. Given their small size, the PBS proteins are ideal candidates for analysis using a top-down proteomics approach. In this study, we further characterized the UTEX 2973 nitrogen starvation-based PBS degradation and re-synthesis response, which included performing a top-down proteomics analysis to detect the proteoforms of ApcA, ApcB, CpcA, CpcB, and CpcD for partial degradation on the protein backbone, or on the PCB pigment molecules. Quantitative changes of NblA were also captured by top-down proteomics and further validated and characterized.

## 2. Materials and Methods

### 2.1. Bacterial Strain and Growth Conditions

*Synechococcus elongatus* UTEX 2973 cultures were grown photoautotrophically in a multicultivator photobioreactor (Photon Systems Instuments, Czech Republic) at 37 °C with 500 μmol photons m^−2^ s^−1^ of light and 5% CO_2_ in BG11 growth medium [36] containing 1.76 M NaNO_3_ (BG11+N).

### 2.2. Nitrogen Starvation Conditions

To initiate nitrogen starvation in UTEX 2973, exponential cultures (OD_730 nm_ = 0.9) grown in multicultivators were harvested, washed twice, and resuspended with BG-11_0_ media (NaNO_3_ was replaced with equimolar amount of NaCl) [16]. The final OD_730 nm_ was adjusted to 0.2 at the onset of the experiment and cultures were placed back in the multicultivators at 37 °C with 500 μmol photons m^−2^ s^−1^ of light and 5% CO_2_. PBS degradation was monitored as a function of time during nitrogen starvation and samples were collected at times 0 h, 0.5 h, 1.5 h, 2 h and 4 h for physiology experiments as well as proteomics. Re-synthesis of PBSs was initiated by the addition of 10 mM NaNO_3_ at the end of 4 h nitrogen starvation. Samples for re-synthesis were collected at the following time points: 0.5 h, 1.5 h, 2 h and 4 h. Cell numbers were calculated by counting for each time point using a Cellometer Auto M10 cell counter (Nexcellom Bioscience, Lawrence, MA, USA). Samples were collected for each time point during PBS degradation and resynthesis from three biological replicates. For nitrogen starvation experiments with *Synechocystis* sp. PCC 6803, cells were grown at 30 °C with a light intensity of 100 μmol photons m^−2^ s^−1^. The sampling period of nitrogen starvation was extended for *Synechocystis* sp. PCC 6803 to capture the process of PBS degradation.

### 2.3. Spectrophotometric Analysis

Absorption spectra (400–750 nm) of whole cells were collected using an Olis DW2000 spectrophotometer (On-Line Instrument Systems, Bogart, GA). The concentrations of phycobilin and chlorophyll *a* (Chl *a*) pigments were calculated using the following equations: 0.139(*A*_620_ − *A*_730_) − 0.0355(*A*_678_ − *A*_730_) = mg/mL and 14.96(*A*_678_ − *A*_730_) − 0.616(*A*_625_ − *A*_730_) = μg/mL, respectively [8,16]. Values were normalized to 1.00 at the start of the experiment to visualize PBS degradation as percent decrease in PC.

### 2.4. Low Temperature (77K) Fluorescence Spectroscopy

Fluorescence emission spectra of whole cells were determined on a Fluoromax-2 fluorometer (Jobin Yvon, Longjumeau, France). Cells were adjusted to 5 μg/mL Chl *a* for all measurements. Chl *a* was excited at 435 nm and phycobilins were excited at 600 nm. Fluorescence emission spectra were normalized by F/F750. Three independent biological replicates (*n* = 3) were measured for both PBS degradation and re-synthesis experiments.

### 2.5. Preparation of PCB Proteins for Mass Spectrometry

Samples were collected at each time point during PBS degradation and re-synthesis from three biological replicates. Cultured cells were frozen until analysis (10^8^–10^9^ cells per sample). After thawing, cells were pelleted at 4500× *g* for 5 min, and then mixed with 900 µL homogenization solution (8 M urea and 1 mM phenylmethylsulfonyl fluoride). Cells were lysed via beating with zirconia beads for 3 min at 4 °C in 2 mL snap cap tubes. A hole was made in the 2 mL tube and the tube was placed in 15 mL tube for spinning at 2500 rpm for 10 min. Beads were allowed to settle after being pipetted up and down. About half of the sample was taken for tryptic digestion for bottom up experiments. Briefly, the sample was first incubated with 10 mM dithiothreitol at 60 °C for 30 min, diluted 10 times with 100 mM ammonium bicarbonate and 1 mM CaCl_2_, then mixed with trypsin at 37 °C for 3 h at 1:50 enzyme:protein ratio. Digested peptides were desalted with C18 solid phase extraction kit (Gilson, Inc., Middleton, WI, USA) and snap frozen until use.

The other half of the sample right after bead beating was further processed for top-down experiments. After incubation at 37 °C for 1 h, the tubes were centrifuged at 100k rpm for 10 min at 4 °C. The supernatant was filtered through 100 k Amicon molecular weight cut-off spin filters (pre-washed with homogenization solution) at 14,000× *g*. The flow-through was then centrifuged with 10 k Amicon spin filters at 14,000× *g*. The retentate was washed with 25 mM ammoinium bicarbonante 3 times and saved for analysis. Protein concentrations were measured by the bicinchoninic acid assay.

### 2.6. Top-Down Mass Spectrometry of PCB Proteins

Extracted proteins (~2.5 µg) were directly loaded onto a Waters NanoAcquity LC equipped with a C2 precolumn and a C2 analytical column (70 cm, inner diameter 100 µm, and column packing with Jupiter 3 µm C2 particles). The LC was connected online to a Thermo LUMOS Orbitrap mass spectrometer. The LC mobile phase was a mixture of water and acetonitrile plus 0.1% formic acid. The gradient was from 10% to 50% acetonitrile over 3h. The electrospray voltage was at 2.4 kV, with source ion transfer tube at 275 °C and S-lens RF level at 60. Precursor MS1 spectra were collected at 240k resolution with AGC target of 1E6 and 3 microscans. The ions with charge states higher than +4 and between m/z of 700–1200 were selected for fragmentation. Dynamic exclusion was enabled for the same precursors within 40 s. Different charge states of the same precursor were also excluded. Precursors with highest charge states and highest intensities were given priority by the instrument control software. MS2 fragmentation spectra were collected at 120k resolution with AGC target 1E6 and 1 microscans. Both electron transfer dissociation (ETD, reaction time 20 ms) and higher-energy collisional dissociation (HCD, collision energy 25%) were acquired with 2 Da isolation windows. As many MS2 spectra as could fit within a cycle time of 20 s were collected between two MS1 precursor spectra. All samples were randomized in order in the sequence for analysis.

PCB proteoforms were identified by MSAlign, an open modification tool for top-down proteomics [37,38], from the resulting LC-MS/MS data. Fragments were matched to the protein database with all *Synechococcus* proteins with 15 ppm tolerance for mass error. Identifications were filtered with an E-value threshold of 1E-10. The abundances of all species detected in MS data were first obtained with ProMex [39]. Custom perl and R scripts were then used to attach abundances from ProMex to the identified target proteoforms from MSAlign. The features were matched based on the criteria of ± 3 Da in mass and similar retention times. The raw abundances from ProMex were normalized so that all runs have the same median abundance values. The reported abundances were further divided by the total cell count for each sample. The average normalized abundance of 3 biological replicates are reported. The mass spectrometry proteomics data have been deposited to the ProteomeXchange Consortium via the PRIDE [40] partner repository with the dataset identifier PXD014590.

### 2.7. Selected Reaction Monitoring (SRM) Experimental Design and Analysis

Peptide Selection and Optimization. Tryptic peptides of NblA were identified with the global bottom-up LC-MS/MS analyses of UTEX 2973 [3]. The suitability of these peptides for SRM analysis was evaluated by the number of observations, ESP predictor [41] and CONSeQuence software [42]. All peptides were further blasted for their uniqueness to the target protein with the final selection of six surrogate peptides (i.e., MLPPLPDFSLSVEQQFDLQK, MAHENIFK, PDFSLSVEQQFDLQK, PPLPDFSLSVEQQFDLQK, LPPLPDFSLSVEQQFDLQK, and EDLEDLFIEVVR). Crude heavy peptides labeled with ^13^C/^15^N on C-terminal lysine and arginine were purchased from New England peptides (Gardner, MA), and they were mixed together to generate a stock solution with each heavy peptide at a nominal concentration of 1 µM (i.e., 1000 fmol/µL). To evaluate the peptide quality and select the best responsive transitions for each peptide, 500 fmol/µL of heavy peptide mixtures were subjected to a TSQ Vantage triple quadrupole mass spectrometer (Thermo Scientific, San Jose, CA, USA) for transition and collision energy optimization by direct infusion at an infusion rate of 500 nL/min. The five best transitions for each target peptide were selected for further analysis and the three most intense transitions were used for target protein quantification [43,44]. Next we employed LC-SRM to further evaluate the six surrogate peptides for LC performance (e.g., the stability of peptide retention time, transition interferences, and endogenous peptide detectability by spiking the heavy internal standards into one test sample. In the end, three surrogate peptides with endogenous detectability (MLPPLPDFSLSVEQQFDLQK, MAHENIFK, and EDLEDLFIEVVR) were selected for configuration of the final panel of assays for reproducible targeted quantification across different conditions. Supplemental Table S1 lists the final panel of SRM assays including peptide transitions and their collision energies.

SRM Instrumental Analysis. Trypsin digested samples that had been stored at −80°C until use were processed as previously described [43,44]. For each sample the digested peptides were diluted to 0.5 µg/µL with 0.1% formic acid (FA) in water containing standards at a nominal concentration of 100 fmol/µL for MAHENIFK and EDLEDLFIEVVR, and 200 fmol/µL for MLPPLPDFSLSVEQQFDLQK. All the samples were analyzed with a nanoACQUITY UPLC^®^ system (Waters Cooperation, Milford, MA) coupled online to a TSQ Vantage triple quadrupole mass spectrometer (Thermo Scientific, San Jose, CA). The LC separation was performed with a reversed-phase capillary column (ACQUITY UPLC BEH 1.7 µm C18 100 µm × 100 mm), which was connected to a chemically-etched 20 µm i.d. fused-silica emitter tip via a Valco stainless steel union. The mobile phase B used was 0.1% FA in 90% acetonitrile (ACN)/10% H_2_O (*v/v*), while the mobile phase A was 0.1% FA in H_2_O. Before running the samples, we optimized the on-column mass loading to maintain high LC reproducibility and endogenous detectability without interferences. A total of 1 µg of peptide mixture was determined to be the optimal loading for achieving higher run-to-run LC reproducibility and maximal sensitivity for detection of endogenous peptides. A total of 2 µL of sample (~1 µg) was injected for LC-SRM analysis with a LC binary gradient: 0.5–10% B for 0.5 min, 10–15% B for 3.5 min, 15–25% B for 21 min, 25–38.5% B for 11 min, 38.5–95% B for 1 min, and then 95% B for 8 min. Typically, the TSQ Vantage mass spectrometer was operated with an ion spray voltage of 2400 ± 100 V, a capillary offset voltage of 35 V, a skimmer offset voltage of –5 V, and a capillary inlet temperature of 220 °C. The tube lens voltages were obtained from automatic tuning and calibration without further optimization. A single scan event was used to monitor all SRM transitions using the following parameters: Q1 and Q3 unit resolution of 0.7 FWHM, Q2 gas pressure of 1.5 mTorr, and scan width of 0.002 *m/z*.

SRM Data Analysis. SRM data acquired on the TSQ Vantage were analyzed using Skyline software [45]. Peak detection and integration were determined based on two criteria: (1) the same retention time, and (2) approximately the same relative SRM peak intensity ratios across multiple transitions between light peptides and heavy peptide standards [44]. All the data were manually inspected to ensure correct peak assignment and peak boundaries. The peak area ratios of endogenous light peptides and their heavy isotope-labeled internal standards (i.e., L/H peak area ratios) were then automatically calculated by Skyline, and the average peak area ratios from all the transitions of 3 biological replicates were used for quantitative analysis of the samples.

## 3. Results

### 3.1. Rapid PBS Degradation and Re-Synthesis in UTEX 2973 during N Starvation

**Figure 1:**
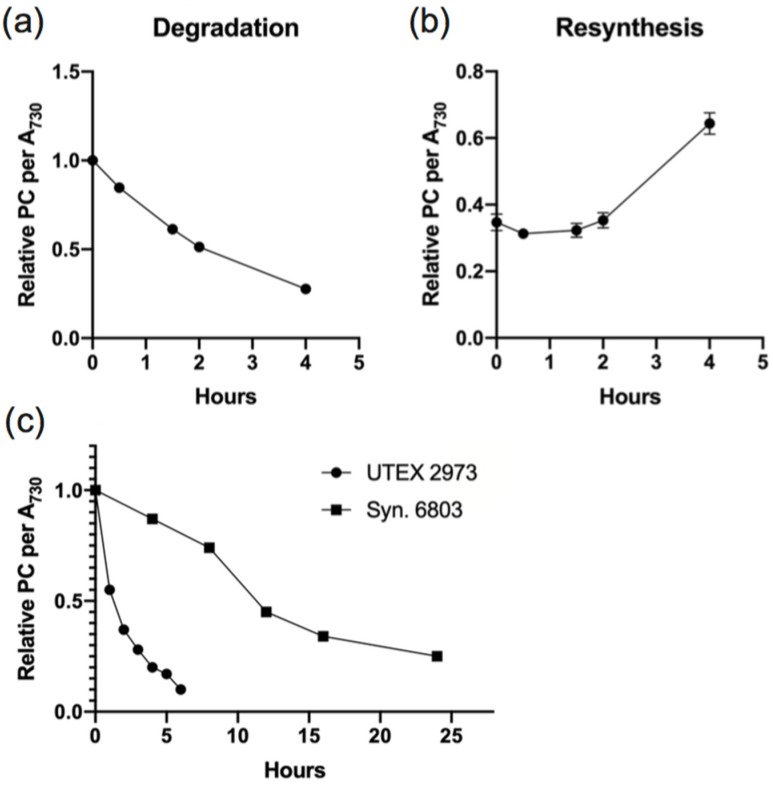
Degradation (**a**) and re-synthesis (**b**) of phycobilisomes (PBSs) in wild type UTEX 2973 during nitrogen depletion and repletion, respectively. Degradation and re-synthesis are calculated by the changes in relative phycocyanin (PC) content per OD_730_ over time. The traces are an average of three independent biological replicates with standard deviation represented as error bars. The error is minor (±0.02) at some time points and therefore not visible in the graphical representation. The raw absorbance values (400–750 nm) were recorded for each of three biological replicates at each time point, and PC concentration was calculated according to [8]. The values at time T0 were normalized to 1.00 to establish the start of the degradation experiment and values for all time points are relative to T0. (**c**) Comparison of PBS degradation in UTEX 2973 and *Synechocystis* sp. PCC 6803. Representative data are shown from a total of three biological replicates in UTEX 2973 and two biological replicates in *Synechocystis* PCC 6803.

UTEX 2973 has been established as one of the more robust cyanobacterial strains with a fast doubling time and increased tolerance to higher light intensities and photodamage. To further evaluate the response of UTEX 2973 to nutrient stress, we subjected exponential cultures of wild type (WT) UTEX 2973 to nitrogen (N) starvation and monitored changes in PC levels by recording the absorption spectra at various time points (Figure 1a). UTEX 2973 was observed to undergo a fast rate of PBS degradation with an approximately 70% decrease in PC per cell within 4 h of N starvation (Figure 1a). Re-addition of N to the cultures resulted in a gradual increase in PC content in the cells as reflected by the absorption spectra indicative of re-synthesis of PBS (Figure 1b). Approximately 60% of PBSs are resynthesized within 4 h of N re-addition.

A comparison of PBS degradation in UTEX 2973 and *Synechocystis* sp. PCC 6803 is shown in Figure 1c to illustrate that the rapid rate of PBS degradation in UTEX 2973 appears to follow first order exponential decay kinetics. Furthermore, the time taken for PBS degradation was much faster than in the previously studied model cyanobacterium *Synechocystis* sp. PCC 6803 with 80% of PBS degraded in 6 h (Figure 1c). In UTEX 2973, the entire process of PBS degradation and re-synthesis can be monitored in 10 h, a process that would typically take more than 5 days in other slower growing cyanobacteria such as *Synechococcus* sp. PCC 7942 and *Synechocystis* sp. PCC 6803 [16,26]. The process of PBS degradation and re-synthesis in UTEX 2973 is dynamic, fast and repeatable, thus enabling the exploration of the immediate consequences of this process in the physiology of the cyanobacterium and the changes that take place on a proteomic level.

### 3.2. Photosystem Levels are Modulated as A Consequence of N Starvation and Re-Addition

**Figure 2:**
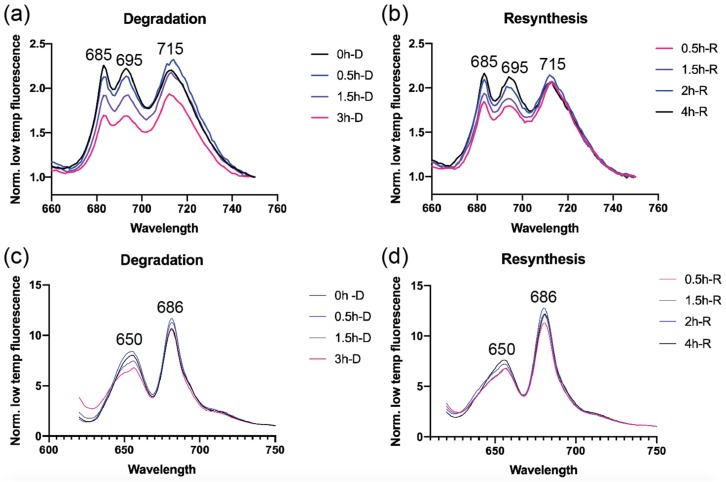
Low temperature (77 K) fluorescence emission spectra of UTEX 2973 with excitation at 435 nm for Chl (**a**,**b**) and at 600 nm for phycobilins (**c**,**d**) taken at different time points during N depletion and repletion. Fluorescence intensities are normalized at 750 nm to highlight differences in the intensities at PSI, PSII, and PC peaks. Peaks are labeled corresponding to the description in the text. Spectra shown are representative data from three independent biological replicates (*n* = 3).

Low temperature fluorescence measurements were taken at various time points during N starvation and re-addition to monitor changes in the photosystems and the energy transfer between PBS and photosystems. Excitation of Chl *a* at 435 nm during PBS degradation showed a gradual decrease in both photosystems I and II (PSI and PSII). PSII showed a more drastic decrease within 1 h of N-starvation compared to PSI as observed by the decrease in 685 nm and 695 nm peaks corresponding to PSII (Figure 2a) [46]. PSI levels measured at 715 nm started decreasing towards the 3 h time point. During re-addition of N, PSI recovered more quickly and was able to return to original levels within 0.5 h, whereas PSII levels gradually increased over 4 h to return to the initial fluorescence peak intensities (Figure 2b). Changes in photosystem levels have been reported previously to occur within 24–48 h of N-starvation and re-addition [19,47]. In UTEX 2973, the changes to photosystems are immediate, occurring within an hour of N starvation.

The effect of N starvation on the photosynthetic electron transport chain was studied by monitoring the P700+ re-reduction kinetics in the presence of DCMU and DBMIB. There was an increased contribution of the cyclic electron flow during N starvation with 25% of total electron flow contributed by the cyclic electron flow, while control (+N) cells maintained a constant 5% cyclic electron flow (Supplementary Figure S1). Therefore, during PBS degradation there is a decrease in PSII to PSI stoichiometry alongside an increase in cyclic electron flow to compensate for the inactive PSII reaction centers.

Excitation of PBS at 600 nm showed that by 3 h after N starvation there is a shift in the PC emission at ~650 nm, indicative of inefficient energy transfer to APC (Figure 2c). This corresponds with a decrease in the fluorescence intensities from the terminal emitter at 686 nm [48]. Addition of N reversed the trends of decreasing fluorescence intensities and rapidly restored the energy transfer between PBS and photosystems (Figure 2d).

### 3.3. Top-Down Proteomics of PBP Proteoforms Linked to PCBs

**Figure 3:**
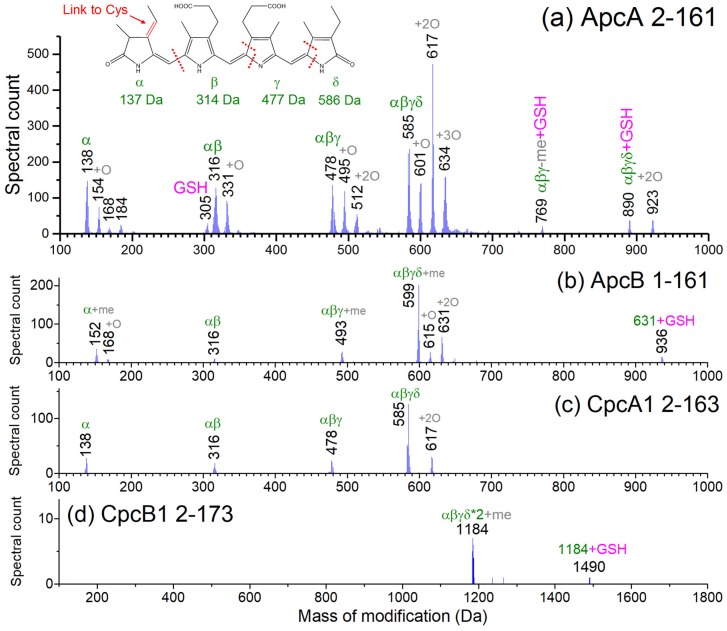
Histogram of detected modifications on full-length (**a**) ApcA, (**b**) ApcB, (**c**) CpcA, and (**d**) CpcB as revealed by top-down proteomics. Spectral counts were calculated based on data from all samples. The modification masses detected on the intact ApcA protein shows clusters of species that can be explained by cleavages between the α, β, γ, δ pyrrole rings in the PCB structure (Figure 4a insert), plus combinations of methylation (me), oxidation (O), and glutathionylation (GSH). Similar but generally fewer types of modifications were observed for ApcB, CpcA with one chromophore each, and CpcB with two chromophores.

In parallel with physiological measurements, samples were collected during nitrogen deprivation and re-addition for proteomics analyses. Multiple PBPs were identified through global LC-MS/MS top-down proteomic analysis, which focuses on direct identification of intact proteins without tryptic digestion. Such data also provides protein modification-based covalent linkage information, in this case to PCBs, which resulted in a range of mass shifts from the unmodified protein sequence predicted from the genome. A core mass shift of ~585 Da was commonly seen for many proteoforms of ApcA, ApcD, and CpcA, which matched to the known mass of PCB [49]. However, we also observed different masses that did not match directly to a single known PCB. In order to identify the unknown modifications, we examined the histogram of the mass shifts (attributed to post-translational modifications (PTMs) but not due to terminal truncation) on the identified MS/MS spectra for the expected members of PBS. ApcA showed a very high variety of PTMs, some of which are shared by other PBPs. However, the masses of the PTMs on ApcA were not randomly distributed and were concentrated at a few specific values around 138, 316, 478, and 617 Da, as shown in Figure 3a. These correlated well with a series of fragments of PCB (Figure 3a inset), mostly with extra modifications of ~16 Da (+15.99 Da for oxidation), via breakage between individual pyrrole rings past the double bonds.

Several other PBPs, including ApcB, ApcD, ApcF, CpcA, and CpcB, were also detected with PCBs attached (Table S2, and representative annotated spectra in Figures S2–S13). Among those, ApcB, CpcA, and CpcB showed a variety of different masses for PTMs, including masses that can be matched to intact PCBs and partially degraded PCBs (Figure 3b–d). Given that numerous PBPs have redox-active protein thiols that are primarily involved in PCB attachment, these PTMs suggest chromophore degradation products that have not been captured in earlier studies. For CpcB, two Cys are known to both be sites for PCB attachment, and therefore the mass of two PCBs were detected on the protein. The beta subunit of PC in *Synechococcus* PCC 6301 (and several other cyanobacterial species) was reported to be specifically methylated on the conserved Asn71 near the Cys with the pigment [49,50], which is believed to be involved in energy transfer [51]. Thus, the different masses of PTMs on ApcB (152, 493, 599 Da) from the rest of the PBPs can be explained by the methylation on the Asn71 (+14.01 Da), which were further supported by the sequence of ions in the data (Figures S5 and S9). For CpcB, Cys83 and Cys154 are modified with PCBs, and Cys110 is likely linked with GSH (glutathione) (Table S2, Figures S11 and S12). ApcA and ApcB each only have only one Cys in their sequences, which means that there is only one thiol site for potential PCB or GSH attachment. Yet the species identified with modification mass of 769, 890, 923 for ApcA, and 936 for ApcB (Figure 3) may be assigned to PCBs plus GSH. Due to incomplete sequence coverage near the Cys in the MS/MS spectra, the GSH might have been linked to residues other than Cys but close to the Cys81. It is also possible that GSH and PCB could be simultaneously linked the Cys81 via unknown chemistry.

**Figure 4:**
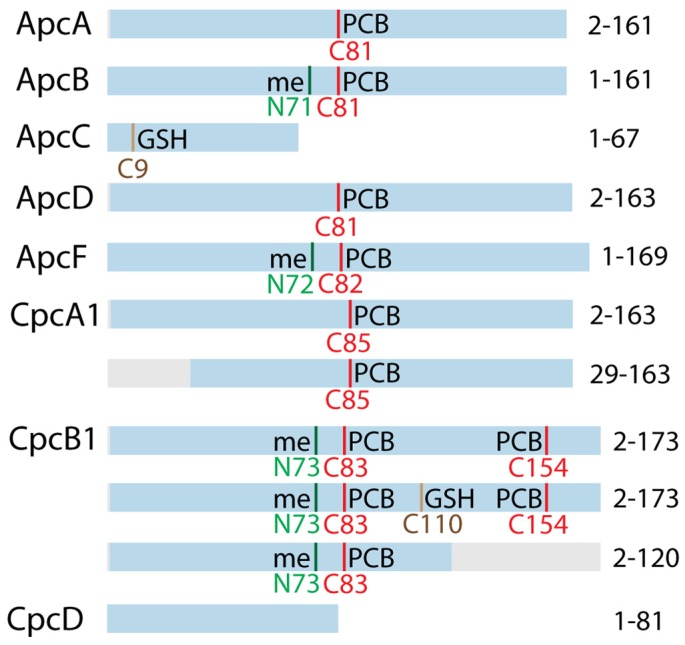
Summary of identified proteoforms of PBS proteins with top-down proteomics, including putatively assigned modifications based on mass shift detected in experimental data in this study and the information in UniProt (PCB—phycocyanobilin (red line), GSH—glutathione (brown line), me—methylation (green line). The blue bars represent the regions of the proteins detected experimentally, the gray bars are the regions that were truncated. PCB is detected at many different masses as discussed in Figure 3. A detailed list of proteoforms is included in Table S2.

A summary of all major proteoforms of PBPs identified is shown in Figure 4. While most PBPs in the list showed primarily full-length proteins, CpcA and CpcB showed some truncated forms with high spectral counts. CpcA had a specific truncation site at residue 29, while CpcB appeared to have a C-terminally truncated proteoform (after residue 120) with one fewer Cys that is linked to PCB. We also detected fragments of CpcD across different regions of the protein with length around 40–60 residues (not shown in Figure 4, listed in Table S2), although their origin is unclear. Because not all detected PBPs were truncated in the same manner or to the same extent, we expect at least part of the truncated forms were generated in vivo, or were biologically related (e.g., regulated degradation under nitrogen starvation). CpcD is annotated as the capping linker protein located on the distal most part of PBS and it is predicted to be degraded first during PBS degradation [52].

### 3.4. Quantitative Changes of Proteins during PBS Degradation and Re-Synthesis

**Figure 5:**
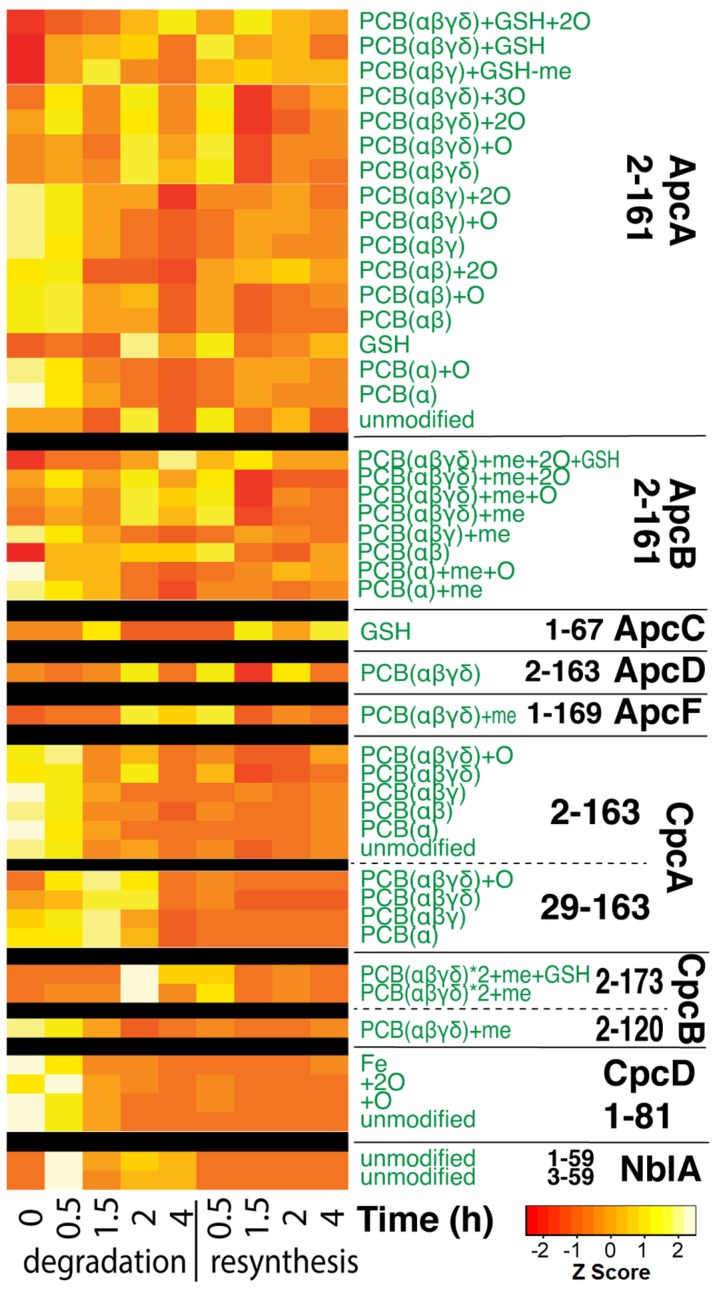
Heatmap showing the relative changes of proteoform abundances of the major phycobiliproteins (PBPs) shown in Figure 3 and Figure 4. Each row represents a proteoform as annotated by the green text, which are grouped by protein. The black text to the right shows the protein name and starting–ending residue numbers. Each column represents the averaged relative abundance of 3 biological replicates at each time point. The color key to the Z score in the heatmap is shown at the lower right bottom.

We looked specifically at abundance changes across the PBS protein components previously identified within the top-down proteomic data. Overall, Figure 5 shows a universal decrease in abundance across all detected PBS protein components, and most modified forms, within the degradation phase. Interestingly, recovery of PBS protein abundances within the re-synthesis phase was more muted and mixed across components (all 3 replicates showed similar trend, Figure S14).

We additionally examined the change of proteoform abundances of all other proteins detected in the experiment. A number of proteins showed responses that correlated to nitrogen starvation. For example, the acetylated form of chemotaxis protein CheY (M744_01170), which is the known active form involved in transmission of sensory signal from chemoreceptors to flagella motors [53], showed significantly higher signal under nitrogen starvation (Figure S15). Two high light inducible proteins (Figure S16) also showed increased abundance at the late stage of nitrogen starvation, followed by decreasing abundance in the re-synthesis stage. These correlated with the changes we observed via Chl *a* fluorescence in the photosystems as a consequence of PBS degradation (Figure 2a). The high light inducible proteins were previously reported to be able to bind and quench chlorophyll and beta-carotene [54]. Most interestingly, however, was the protein NblA (last two rows in Figure 5), which correlated closely with PBS degradation. NblA was upregulated specifically during the degradation time points and its abundance decreased during the re-synthesis phase. NblA is a proteolysis adaptor that triggers PBS degradation, and NblA transcripts have been shown previously to be induced during N starvation [55]. These data highlight an induction at the translational level as well.

### 3.5. The NblA Protein Undergoes >50-Fold Induction under N Starvation

**Figure 6:**
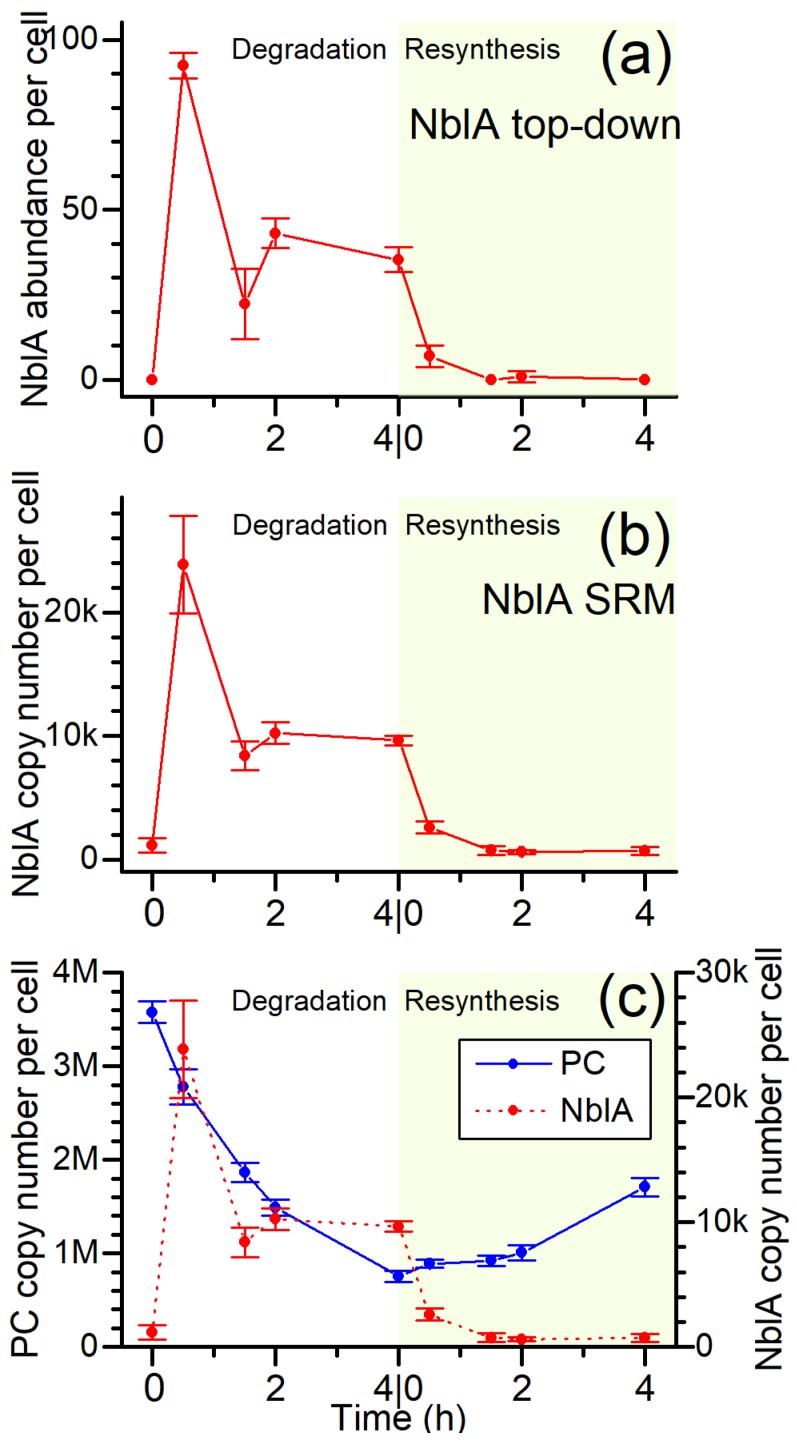
Change in NblA concentration over time as measured by (**a**) top-down proteomics and (**b**) selected reaction monitoring (SRM) bottom-up proteomics. Standard deviations of the measurements on 3 biological replicates are shown as error bars for the sample (red curve with filled circles). In bottom-up proteomics, the copy number of a NblA peptide MAHENIFK was calculated based on the mass spectrometry signal relative to an isotope labeled peptide as internal standard. In top-down proteomics, the mass spectrometry signal from intact NblA protein was directly used for label-free quantitation. Both methods showed a consistent result of spiking NblA concentration at the beginning of the degradation process (white background), and decreased concentration in the re-synthesis phase (green background). (**c**) Ratio of PC and NblA copy numbers during PBS degradation and re-synthesis. Number of NblA molecules was quantified by bottom up proteomics as in (b). PC abundance was calculated from absorbance values collected in Figure 1a,b. NblA and PC per cell were calculated by normalizing the abundance to cell number obtained by cell counting for each of the three biological replicates at every time point. Moles of PC were calculated using the formula provided in [9].

NblA is a small 7 KDa protein that has been previously recognized as the proteolysis adaptor for PBS degradation [26,56]. Despite the important role of NblA in PBS degradation, NblA levels have not been quantified during the degradation process. We looked specifically within the proteomics data for NblA identifications and observed the full length NblA (1–59) along with another proteoform of NblA missing the first two amino acids (3–59). No PTMs were detected on NblA (other than limited Met oxidation). Plotting the normalized protein abundance across the degradation and re-synthesis time points (Figure 6a) resulted in a clear quantitative spike and stabilization peak prior to re-addition of N and the return to undetectable levels.

These results were verified quantitatively with a well-established targeted bottom-up selective reaction monitoring (SRM) proteomics workflow to quantify the change in NblA concentration based on the abundance of selected NblA peptides relative to spiked peptide standards with heavy isotope label (see Figure 6b, details in Materials and Methods). We obtained the near exact pattern of abundance changes for NblA across the time course with >50-fold increase in NblA within 0.5 h of N depletion. The agreement in the quantitation results between the two methods adds validity to using label-free top-down proteomics to probe relatively large changes in protein concentration at the intact proteoform level. The rapid increase of NblA at 0.5 h is most likely a direct response of the cells sensing a lack of nutrients. Interestingly, the NblA concentration dropped at 1.5 h, recovered slightly at 2 h, and plateaued at similar levels to 4 h before decreasing to undetectable background levels when the nitrogen was replenished. Although it is possible that the limited time points collected did not capture the full profile of the NblA response, the “bimodal” curve suggests an underlying regulatory mechanism enabling the expression of NblA to repress PBS formation until sufficient resources, i.e., nitrogen, are available to signal re-synthesis of PBS. For example, the unmodified form of an unknown protein (M744_12535), which blasted to nitrogen starvation response protein, was not detected at significant abundance in the first hour but rapidly started increasing at 2 h (Figure S17). Because the timing matched to the small rebound of NblA at 2 h, we suspect it may be linked to some signaling pathways of PBS degradation. Several other proteoforms of this protein (Figure S17, with annotated spectra in Figures S18–S21) were also detected, yet they show different patterns, suggesting the modifications could affect the biological activity.

We compared PC and NblA concentrations in cells and observed that the quantitative expression profile of NblA correlated well with the PBS degradation rate. Cells maintained approximately 10,000 copies NblA during N starvation. The initial spike in NblA to 25,000 copies at 0.5 h was probably the initial trigger for PBS degradation to compensate for increased number PBS complexes. Based on these calculations we have estimated between 75–220 PC molecules to be associated with one NblA on average during the degradation process (Figure 6c).

## 4. Discussion

In cyanobacteria, light harvesting is accomplished by the membrane-extrinsic PBS antenna complexes. The large size of PBSs, accounting for at least 50% of total soluble protein, make them ideal substrate reserves for cells under challenging environmental conditions, potentially providing amino acids for other cellular processes. When starved for macronutrients such as nitrogen, the degradation of PBS results in a bleaching phenotype (chlorosis), in which the cells lose PC content and become yellow. Upon the re-addition of nitrogen, the cells recover and regain their blue-green color. This phenomenon has been extensively studied in certain cyanobacterial strains including *Synechococcus* sp. PCC 7942 and *Synechocystis* sp. PCC 6803. However, PBS degradation is a lengthy process in these strains, extending over a period of days. We have found that in the fast-growing cyanobacterium UTEX 2973, the process of PBS degradation and re-synthesis can be monitored over a time frame of hours (Figure 1). This compressed time line allows for the study of PBS degradation and re-synthesis as a rapid and synchronized process. The rate of PBS degradation follows an exponential decay and its relevance is evident in the correlation between the NblA copies and PC numbers in the cell, wherein the rate is dependent on the amount of PC present in the cell during N starvation (Figure 6c). A drop in the NblA levels at 1.5 h suggests NblA proteins are also degraded during PBS degradation. As the PBS concentration is decreases over time, lower levels of NblA are maintained within the cell, as seen by the plateau in NblA concentration observed 2 h into N depletion (Figure 6).

Changes to photosystems have been reported previously in prolonged nutrient limitation experiments but our results indicate that PSII also undergoes immediate changes within 1h of the initiation of N starvation, possibly to prevent photodamage. There is a decrease in PSII to PSI stoichiometry, and consequently the contribution of cyclic electron flow (via PSI) increases from 5% to 25% (Figure S1). These downstream changes to photosystems are consistent with previous studies; however, they occur on a much faster timescale in UTEX 2973 [25,47].

Despite decades of research, many details on the molecular mechanism of PBS degradation and re-synthesis remain elusive. The popular model of degradation describes a process in which the PBS rods are degraded starting at the distal ends and continuing until both the rods and core are truncated. However, the exact mechanism behind this model of PBP degradation is unknown. In the current work, we used top-down proteomics and detected a number of modifications and proteoforms of the PBPs throughout the time course that may play a role in the degradation process (Figure 3 and Figure 4).

While many different proteoforms were detected, it is unclear how the “fragments” were generated. Because the major PBPs were not uniformly degraded in the same manner, we suspect, at least partially, that they may have been produced through specific biological reactions and/or photochemistry in vivo. Truncated forms of CpcA and CpcB identified using the top-down approach correlated with regions of the proteins that have been shown to bind NblA [56,57]. The presence of glutathione and the large number of oxidized forms, in particular for ApcA, implies oxidative damage on the PCBs.

The putative assignments of modifications on the PBP proteoforms are solely based on mass from MS data, and it is possible that there were combinations of other PTMs, or even PCBs other than PC, with similar masses. Despite the high resolution of the MS, the large number of isotope peaks (mostly from natural ^13^C1) of intact proteins makes it challenging to precisely define the monoisotopic mass [30], sometimes leading to mass error at intervals of 1 Da. In addition, potential combinations of other PTMs such as deamidation (+0.98 Da) or disulfide bonds (+2.02 Da) can further complicate assignments. Our global bottom-up analysis (data not shown) did reveal several types of modifications, mostly oxidation and a few cases of methylation for the PBPs of interest. However, due to the use of a standard bottom-up workflow that reduced and alkylated the thiol groups, few PCB modifications expected on Cys residues were preserved and confidently identified. A previous quantitative bottom-up proteomics study showed that the changes of PBP concentrations under 24 h N depletion in *Synechocystis* sp. PCC 6803 were different for each subunit [58]. There were minimal changes for ApcA, ApcB, ApcC, ApcD, and ApcF; ~10% decrease of CpcD; ~50–70% decrease for CpcA and CpcB. A more recent study also examined the re-synthesis phase [47] and the resuscitation of the PBPs only became noticeable after 8 h at the 24 h sampling point. It is possible that the fold change of these potential degradation intermediates in UTEX 2973 were too small to be captured with the label-free top-down proteomics method. Several other PBPs (ApcE, CpcC, CpcG) were not detected likely due to their association with the membrane or due to their larger molecular weight, thereby not meeting the cut off for top down proteomics.

Additional bottom-up experiments targeting the PTMs on Cys may better capture the exact modification sites, and the information can then be integrated to improve the interpretation of top-down data [59]. Nonetheless, the top-down data offered insights into potential degradation intermediates of protein-linked PCBs that can be further characterized in a targeted manner. This is the first study to have detected these PCB linked protein modifications that could be attributed to the immediate spectroscopic changes we see during N starvation.

Future top-down studies using newly developed MS-compatible detergent [60] may improve the detection and coverage of the complete family of PBPs, particularly those that are associated with the membrane such as ApcE and other larger proteins that are difficult to solubilize. These undetected proteins also contribute to the change of PBS structure and subsequent decrease of light absorption. Alternatively, there could be other pathways that are able to rapidly control the absorption of PBS in UTEX 2973 without the need of covalently degrading the PCBs or PBPs. The mechanism of PBS degradation likely involves many other components that remain to be discovered. The top-down data provided valuable qualitative insights on some of the potential intermediates of the degraded proteoforms, which could serve as new targets for future studies. Quantitative analysis of protein abundances revealed interesting changes to NblA, along with a few other proteins that may be directly linked to the regulation of PBS degradation. With the advancing top-down proteomics technology, we hope to obtain deeper coverage of the changes to the truncated and partially degraded PBPs, including the membrane associated proteins, to complete our understanding of this process.

NblA is a small 7 kDa protein that has been described as a proteolysis adapter that binds to PBS and targets them to Clp proteasome system. In vitro and mutational studies have identified binding locations for NblA and PC subunits. However, the mechanism of how a small 7kDa protein triggers the degradation of <7MDa protein complex is unclear. It is speculated to be co-degraded with PBS in a ubiquitin-like manner. Another popular hypothesis is that it results in the disassembly of PBS. One of the outstanding questions that can help increase our understanding of this tightly regulated phenomenon is the number of copies of the NblA protein during N starvation. Both from top-down as well as bottom-up data we showed a >50-fold increase in the expression levels of NblA. Absolute quantitation of NblA using heavy isotope labeled peptide standards showed a maximum of ~25,000 copies of NblA per cell (Figure 6a,b). The tight regulation of NblA is evident from the immediate repression in NblA levels within 0.5 h of addition of N. Even though the number of time points are limited to see the full trend, it appears that cells seem to maintain NblA at approximately 10,000 copies after the initial spike at 0.5 h post N starvation. The drop in NblA levels at the 1.5 h time point suggests that NblA proteins are co-degraded during PBS degradation similar to ubiquitin (Figure 6).

The expression levels of NblA correlated well with decreasing PC concentration during N starvation. From the spectroscopic determination of PC molecules and comparison to the NblA copy number, we have estimated an association of 75–220 PCs per NblA protein (Figure 6c). It has been reported that UTEX 2973 has lower PC content than its closest relative *Synechococcus* sp. PCC 7942 [61]. Given that there are more than 100 PCs in a bicylindrical PBS complex, we expect this number to be lower than 100 in UTEX 2973. Therefore, during PBS degradation one NblA has the potential to associate with 1–2 PBS complexes. Based on these results it can be hypothesized that NblA associates with PBSs to destabilize PBS complexes before becoming degraded by a Clp protease. NblA proteins potentially undergo degradation as well to maintain lower levels of NblA as the PBS concentration decreases during the time course. This can be observed by the plateau in NblA copies observed from 2 h into N starvation. Future in vivo studies to track the fate of NblA will provide more insights into how NblA triggers degradation of PBS complexes and the role of Clp proteasome in this process.

## 5. Conclusions

In cyanobacteria, the PBS light harvesting antenna complex can account for 50% or more of soluble cellular protein. Given their large size, these pigment-protein complexes are surprisingly adaptable and responsive to changes in environmental conditions. Cyanobacteria are found in a variety of challenging habitats, and the ability to degrade PBS complexes in response to macronutrient depletion aids in cell survival by providing an amino acid reservoir. The regulation and mechanism of PBS degradation in the fast-growing cyanobacterium *Synechococcus* UTEX 2973 was explored by a combination of physiological and proteomic analyses. Our results showed that PBS degradation is very rapid in this strain compared to other model cyanobacteria, with 80% of PBSs per cell degraded within six hours under optimal conditions. Together, degradation and re-synthesis can be monitored over a period of 10 h. Proteomic analysis during a time course of PBS degradation and re-synthesis revealed multiple previously unidentified proteoforms of the PBPs with partially degraded phycocyanobilin (PCB) pigments. Additionally, NblA, a small proteolysis adaptor that is essential in PBS degradation, was characterized and validated with targeted mass spectrometry. For the first time, NblA levels were quantified during PBS degradation and re-synthesis, and results showed that levels rose from ~0 to 25,000 copies per cell within 30 min of nitrogen depletion. These data suggest a model in which approximately one NblA molecule associates with and aids in the degradation of each PBS during the degradation process.

## Supplementary Materials

The following are available online at https://www.mdpi.com/2218-273X/9/8/374/s1, Figures S1–S21; Table S1: Transitions used for targeted SRM quantitation of NblA, Table S2: Identified proteoforms of PBS proteins with top-down proteomics workflow.

## References

[1] Bogorad L. (1975). Phycobiliproteins and complementary chromatic adaptation. Annu. Rev. Plant Physiol..

[2] Gantt E., Bryant D.A. (1994). Supramolecular membrane organization. The Molecular Biology of Cyanobacteria.

[3] Yu J., Liberton M., Cliften P.F., Head R.D., Jacobs J.M., Smith R.D., Koppenaal D.W., Brand J.J., Pakrasi H.B. (2015). *Synechococcus elongatus* UTEX 2973, a fast growing cyanobacterial chassis for biosynthesis using light and CO_2_. Sci. Rep..

[4] Ungerer J., Lin P.C., Chen H.Y., Pakrasi H.B. (2018). Adjustments to photosystem stoichiometry and electron transfer proteins are key to the remarkably fast growth of the cyanobacterium *Synechococcus elongatus* UTEX 2973. mBio.

[5] Adir N. (2005). Elucidation of the molecular structures of components of the phycobilisome: Reconstructing a giant. Photosynth. Res..

[6] Adir N., Dines M., Klartag M., McGregor A., Melamed-Frank M. (2006). Assembly and disassembly of phycobilisomes. Complex Intracellular Structures in Prokaryotes.

[7] Grossman A.R., Schaefer M.R., Chiang G.G., Collier J.L. (1993). The phycobilisome, a light-harvesting complex responsive to environmental conditions. Microbiol. Mol. Biol. Rev..

[8] Arnon D.I., McSwain B.D., Tsujimoto Y., Wada K. (1974). Photochemical activity and components of membrane preparetion from blue-green algea.I. Coexistance of two photosystems in relation to chlorophyll *a* and removal of phycocyanin. Biochim. Biophys. Acta Bioenerg..

[9] Myers J., Graham J.R., Wang R.T. (1980). Light harvesting in *Anacystis nidulans* studied in pigment mutants. Plant Physiol..

[10] Arteni A.A., Ajlani G., Boekema E.J. (2009). Structural organisation of phycobilisomes from *Synechocystis* sp. strain PCC 6803 and their interaction with the membrane. BBA Bioenerg..

[11] Zhang J., Ma J., Liu D., Qin S., Sun S., Zhao J., Sui S.F. (2017). Structure of phycobilisome from the red alga *Griffithsia pacifica*. Nature.

[12] Calzadilla P.I., Muzzopappa F., Setif P., Kirilovsky D. (2019). Different roles for ApcD and ApcF in *Synechococcus elongatus* and *Synechocystis* sp. PCC 6803 phycobilisomes. Biochim. Biophys. Acta Bioenerg..

[13] Collier J.L., Herbert S.K., Fork D.C., Grossman A.R. (1994). Changes in the cyanobacterial photosynthetic apparatus during acclimation to macronutrient deprivation. Photosynth. Res..

[14] Sauer J., Schreiber U., Schmid R., Volker U., Forchhammer K. (2001). Nitrogen starvation-induced chlorosis in *Synechococcus* PCC 7942. Low-level photosynthesis as a mechanism of long-term survival. Plant Physiol..

[15] Schwarz R., Forchhammer K. (2005). Acclimation of unicellular cyanobacteria to macronutrient deficiency: Emergence of a complex network of cellular responses. Microbiology.

[16] Collier J.L., Grossman A.R. (1992). Chlorosis induced by nutrient deprivation in *Synechococcus* sp. strain PCC 7942: Not all bleaching is the same. J. Bacteriol..

[17] Duke C.S., Cezeaux A., Allen M.M. (1989). Changes in polypeptide composition of *Synechocystis* sp. strain 6308 phycobilisomes induced by nitrogen starvation. J. Bacteriol..

[18] Lau R.H., MacKenzie M.M., Doolittle W.F. (1977). Phycocyanin synthesis and degradation in the blue-green bacterium *Anacystis nidulans*. J. Bacteriol..

[19] Li H., Sherman L.A. (2002). Characterization of *Synechocystis* sp. strain PCC 6803 and ∆*nblA* mutants under nitrogen-deficient conditions. Arch. Microbiol..

[20] Murton J., Nagarajan A., Nguyen A.Y., Liberton M., Hancock H.A., Pakrasi H.B., Timlin J.A. (2017). Population-level coordination of pigment response in individual cyanobacterial cells under altered nitrogen levels. Photosynth. Res..

[21] Schwarz R., Grossman A.R. (1998). A response regulator of cyanobacteria integrates diverse environmental signals and is critical for survival under extreme conditions. Proc. Natl. Acad. Sci. USA.

[22] Dolganov N., Grossman A.R. (1999). A polypeptide with similarity to phycocyanin alpha-subunit phycocyanobilin lyase involved in degradation of phycobilisomes. J. Bacteriol..

[23] Van Waasbergen L.G., Dolganov N., Grossman A.R. (2002). nblS, a gene involved in controlling photosynthesis-related gene expression during high light and nutrient stress in *Synechococcus elongatus* PCC 7942. J. Bacteriol..

[24] Forchhammer K., Schwarz R. (2019). Nitrogen chlorosis in unicellular cyanobacteria—A developmental program for surviving nitrogen deprivation. Environ. Microbiol..

[25] Collier J.L., Grossman A.R. (1994). A small polypeptide triggers complete degradation of light-harvesting phycobiliproteins in nutrient-deprived cyanobacteria. EMBO J..

[26] Baier A., Winkler W., Korte T., Lockau W., Karradt A. (2014). Degradation of phycobilisomes in *Synechocystis* sp. PCC 6803: Evidence for essential formation of an NblA1/NblA2 heterodimer and its codegradation by a Clp protease complex. J. Biol. Chem..

[27] Cox J., Hein M.Y., Luber C.A., Paron I., Nagaraj N., Mann M. (2014). Accurate proteome-wide label-free quantification by delayed normalization and maximal peptide ratio extraction, termed MaxLFQ. Mol. Cell. Proteom..

[28] Rauniyar N., Yates J.R. (2014). Isobaric labeling-based relative quantification in shotgun proteomics. J. Proteome Res..

[29] Schubert O.T., Röst H.L., Collins B.C., Rosenberger G., Aebersold R. (2017). Quantitative proteomics: Challenges and opportunities in basic and applied research. Nat. Protoc..

[30] Schaffer L.V., Millikin R.J., Miller R.M., Anderson L.C., Fellers R.T., Ge Y., Kelleher N.L., LeDuc R.D., Liu X., Payne S.H. (2019). Identification and quantification of proteoforms by mass spectrometry. Proteomics.

[31] Toby T.K., Fornelli L., Kelleher N.L. (2016). Progress in top-down proteomics and the analysis of proteoforms. Ann. Rev. Anal. Chem..

[32] Skinner O.S., Haverland N.A., Fornelli L., Melani R.D., Do Vale L.H.F., Seckler H.S., Doubleday P.F., Schachner L.F., Srzentić K., Kelleher N.L. (2017). Top-down characterization of endogenous protein complexes with native proteomics. Nat. Chem. Biol..

[33] Huang H., Lin S., Garcia B.A., Zhao Y. (2015). Quantitative proteomic analysis of histone modifications. Chem. Rev..

[34] Ntai I., Kim K., Fellers R.T., Skinner O.S., Smith A.D.T., Early B.P., Savaryn J.P., LeDuc R.D., Thomas P.M., Kelleher N.L. (2014). Applying label-free quantitation to top down proteomics. Anal. Chem..

[35] Zhang J., Guy M.J., Norman H.S., Chen Y.C., Xu Q., Dong X., Guner H., Wang S., Kohmoto T., Young K.H. (2011). Top-down quantitative proteomics identified phosphorylation of cardiac troponin I as a candidate biomarker for chronic heart failure. J. Proteome Res..

[36] Allen M.M. (1968). Simple conditions for growth of unicellular blue-green algae on plates. J. Phycol..

[37] Kou Q., Xun L., Liu X. (2016). TopPIC: A software tool for top-down mass spectrometry-based proteoform identification and characterization. Bioinformatics.

[38] Liu X., Sirotkin Y., Shen Y., Anderson G., Tsai Y.S., Ting Y.S., Goodlett D.R., Smith R.D., Bafna V., Pevzner P.A. (2012). Protein identification using top-down spectra. Mol. Cell. Proteom..

[39] Park J., Piehowski P.D., Wilkins C., Zhou M., Mendoza J., Fujimoto G.M., Gibbons B.C., Shaw J.B., Shen Y., Shukla A.K. (2017). Informed-Proteomics: Open-source software package for top-down proteomics. Nat. Methods.

[40] Perez-Riverol Y., Csordas A., Bai J., Bernal-Llinares M., Hewapathirana S., Kundu D.J., Inuganti A., Griss J., Mayer G., Eisenacher M. (2019). The PRIDE database and related tools and resources in 2019: Improving support for quantification data. Nucleic Acids Res..

[41] Fusaro V.A., Mani D.R., Mesirov J.P., Carr S.A. (2009). Prediction of high-responding peptides for targeted protein assays by mass spectrometry. Nat. Biotechnol..

[42] Eyers C.E., Lawless C., Wedge D.C., Lau K.W., Gaskell S.J., Hubbard S.J. (2011). CONSeQuence: Prediction of reference peptides for absolute quantitative proteomics using consensus machine learning approaches. Mol. Cell. Proteom..

[43] Shi T., Su D., Liu T., Tang K., Camp D.G., Qian W.J., Smith R.D. (2012). Advancing the sensitivity of selected reaction monitoring-based targeted quantitative proteomics. Proteomics.

[44] Shi T., Sun X., Gao Y., Fillmore T.L., Schepmoes A.A., Zhao R., He J., Moore R.J., Kagan J., Rodland K.D. (2013). Targeted quantification of low ng/mL level proteins in human serum without immunoaffinity depletion. J. Proteome Res..

[45] MacLean B., Tomazela D.M., Shulman N., Chambers M., Finney G.L., Frewen B., Kern R., Tabb D.L., Liebler D.C., MacCoss M.J. (2010). Skyline: An open source document editor for creating and analyzing targeted proteomics experiments. Bioinformatics.

[46] Andrizhiyevskaya E.G., Chojnicka A., Bautista J.A., Diner B.A., van Grondelle R., Dekker J.P. (2005). Origin of the F685 and F695 fluorescence in photosystem II. Photosynth. Res..

[47] Spät P., Klotz A., Rexroth S., Maček B., Forchhammer K. (2018). Chlorosis as a developmental program in cyanobacteria: The proteomic fundament for survival and awakening. Mol. Cell. Proteom..

[48] Yu J., Wu Q., Mao H., Zhao N., Vermaas W.F. (1999). Effects of chlorophyll availability on phycobilisomes in *Synechocystis* sp. PCC 6803. IUBMB Life.

[49] Shen G., Schluchter W.M., Bryant D.A. (2008). Biogenesis of Phycobiliproteins: I. *cpcS-I* and *cpcU* mutants of the cyanobacterium *Synechococcus* sp. PCC 7002 define a heterodimeric phyococyanobilin lyase specific for β-phycocyanin and allophycocyanin subunits. J. Biol. Chem..

[50] Klotz A.V., Glazer A.N. (1987). gamma-N-methylasparagine in phycobiliproteins. Occurrence, location, and biosynthesis. J. Biol. Chem..

[51] Swanson R.V., Glazer A.N. (1990). Phycobiliprotein methylation: Effect of the γ-N-methylasparagine residue on energy transfer in phycocyanin and the phycobilisome. J. Mol. Biol..

[52] De Lorimier R., Bryant D.A., Stevens S.E. (1990). Genetic analysis of a 9 kDa phycocyanin-associated linker polypeptide. Biochim. Biophys. Acta.

[53] Paul K., Nieto V., Carlquist W.C., Blair D.F., Harshey R.M. (2010). The c-di-GMP binding protein YcgR controls flagellar motor direction and speed to affect chemotaxis by a “backstop brake” mechanism. Mol. Cell.

[54] Komenda J., Sobotka R. (2016). Cyanobacterial high-light-inducible proteins—Protectors of chlorophyll–protein synthesis and assembly. Biochim. Biophys. Acta Bioenerg..

[55] Richaud C., Zabulon G., Joder A., Thomas J.C. (2001). Nitrogen or sulfur starvation differentially affects phycobilisome degradation and expression of the *nblA* gene in *Synechocystis* strain PCC 6803. J. Bacteriol..

[56] Nguyen A.Y., Bricker W.P., Zhang H., Weisz D.A., Gross M.L., Pakrasi H.B. (2017). The proteolysis adaptor, NblA, binds to the N-terminus of beta-phycocyanin: Implications for the mechanism of phycobilisome degradation. Photosynth. Res..

[57] Bienert R., Baier K., Volkmer R., Lockau W., Heinemann U. (2006). Crystal structure of NblA from *Anabaena* sp. PCC 7120, a small protein playing a key role in phycobilisome degradation. J. Biol. Chem..

[58] Spät P., Maček B., Forchhammer K. (2015). Phosphoproteome of the cyanobacterium *Synechocystis* sp. PCC 6803 and its dynamics during nitrogen starvation. Front. Microbiol..

[59] Dai Y., Shortreed M.R., Scalf M., Frey B.L., Cesnik A.J., Solntsev S., Schaffer L.V., Smith L.M. (2017). Elucidating *Escherichia coli* proteoform families using intact-mass proteomics and a global PTM discovery database. J. Proteome Res..

[60] Brown K.A., Chen B., Guardado-Alvarez T.M., Lin Z., Hwang L., Ayaz-Guner S., Jin S., Ge Y. (2019). A photocleavable surfactant for top-down proteomics. Nat. Methods.

[61] Ungerer J., Wendt K.E., Hendry J.I., Maranas C.D., Pakrasi H.B. (2018). Comparative genomics reveals the molecular determinants of rapid growth of the cyanobacterium *Synechococcus elongatus* UTEX 2973. Proc. Natl. Acad. Sci. USA.

