# Supplementary material for: Proteomic Insights into Phycobilisome Degradation, A Selective and Tightly Controlled Process in The Fast-Growing Cyanobacterium Synechococcus elongatus UTEX 2973
Source: Biomolecules. 2019 Aug 16;9(8):374. doi: 10.3390/biom9080374 (PMC6722726; doi:10.3390/biom9080374)
Supplement: Supplementary file 1 [file biomolecules-09-00374-s001.pdf]

Supplementary Materials:

Proteomic Insights into Phycobilisome Degradation, A Selective and Tightly Controlled Process in The Fast-Growing Cyanobacterium *Synechococcus elongatus* UTEX 2973

Aparna Nagarajan [1,†], Mowei Zhou [2], Amelia Y. Nguyen [1,‡], Michelle Liberton [1], Komal Kedia [3,§], Tujin Shi [3], Paul Piehowski [3], Anil Shukla [3], Thomas L. Fillmore [3], Carrie Nicora[3], Richard D. Smith [3], David W. Koppenaal [3], Jon M. Jacobs [3] and Himadri B. Pakrasi [1,*]

[1] Department of Biology, Washington University, St. Louis, MO 63130, USA
[2] Environmental and Molecular Sciences Laboratory, Pacific Northwest National Laboratory, Richland, WA 99354, USA
[3] Biological Sciences Division, Pacific Northwest National Laboratory, Richland, WA 99354, USA
* Correspondence: pakrasi@wustl.edu; Tel.: +314-935-6853
† Current address: Living Ink Technologies, Aurora, CO 80045, USA
‡ Current address: U.S. EPA Office of Chemical Safety and Pollution Prevention, Washington, DC 20460, USA
§ Current address: Merck, West Point, PA 19486, USA

Table of Contents

Table S1. Transitions used for targeted SRM quantitation of NblA.

Table S2. Identified proteoforms of PBS proteins with top-down proteomics workflow.

Figure S1. Cyclic electron flow in UTEX 2973 under nitrogen depletion (-N) and repletion (+N).

Figure S2. Example data for identified ApcA proteoform with one PCB on Cys81.

Figure S3. Example data for identified ApcA proteoform with modification mass of 890Da.

Figure S4. Example data for identified ApcA proteoform with modification mass of 923Da.

Figure S5. Example data for identified ApcB proteoform with one PCB on Cys81, and one methyl on N71.

Figure S6. Example data for identified ApcB proteoform with modification mass of 936Da.

Figure S7. Example data for identified ApcC proteoform with glutathione on Cys9.

Figure S8. Example data for identified ApcD proteoform with one PCB on Cys8.

Figure S9. Example data for identified ApcF proteoform with one PCB on Cys82, and one methyl on N72.

Figure S10. Example data for identified CpcA proteoform with one PCB on Cys85.

Figure S11. Example data for identified CpcB proteoform with methyl on N73, two PCBs on Cys83 and Cys154.

Figure S12. Example data for identified CpcB proteoform with methyl on N73, two PCBs on Cys83 and Cys154, and glutathione on Cys110.

Figure S13. Example data for the truncated CpcB proteoform (residue 2-120) with methyl on N73, one PCs on Cys83.

Figure S14. Heatmap for abundance changes of proteoforms including all replicates.

Figure S15. Abundance change across time points for M744_01170 chemotaxis protein CheY.

Figure S16. Abundance change across time points for two highlight inducible proteins.

Figure S17. Abundance change across time points for the major proteoforms of M744_12535.

Figure S18. Example data for the nitrogen starvation response protein M744_12535 without modification.

Figure S19. Example data for the nitrogen starvation response protein M744_12535 with +48.0 Da.

Figure S20. Example data for the nitrogen starvation response protein M744_12535 with 79.96 Da modification on Tyr111.

Figure S21. Example data for the identified nitrogen starvation response protein M744_12535 with glutathionation on Cys113.

Q1 mass	Q3 mass	Collision energy	Identity
495.247	394.208	13	MAHENIFK_light
495.247	583.229	17	MAHENIFK_light
495.247	650.351	14	MAHENIFK_light
495.247	787.41	13	MAHENIFK_light
495.247	858.447	14	MAHENIFK_light
499.254	398.216	13	MAHENIFK_heavy
499.254	583.229	17	MAHENIFK_heavy
499.254	658.365	14	MAHENIFK_heavy
499.254	795.424	13	MAHENIFK_heavy
499.254	866.461	14	MAHENIFK_heavy
738.883	502.298	20	EDLEDLFIEVVR_light
738.883	615.382	21	EDLEDLFIEVVR_light
738.883	875.535	24	EDLEDLFIEVVR_light
738.883	990.562	21	EDLEDLFIEVVR_light
738.883	1119.604	20	EDLEDLFIEVVR_light
738.883	1232.689	22	EDLEDLFIEVVR_light
743.887	512.307	20	EDLEDLFIEVVR_heavy
743.887	625.391	21	EDLEDLFIEVVR_heavy
743.887	885.543	24	EDLEDLFIEVVR_heavy
743.887	1000.57	21	EDLEDLFIEVVR_heavy
743.887	1129.613	20	EDLEDLFIEVVR_heavy
743.887	1242.697	22	EDLEDLFIEVVR_heavy
778.068	696.693	5	MLPPLPDFSLSVEQQFDLQK_light
778.068	778.409	15	MLPPLPDFSLSVEQQFDLQK_light
778.068	915.461	6	MLPPLPDFSLSVEQQFDLQK_light
778.068	1044.536	13	MLPPLPDFSLSVEQQFDLQK_light
778.068	1134.579	20	MLPPLPDFSLSVEQQFDLQK_light
778.068	1421.727	25	MLPPLPDFSLSVEQQFDLQK_light
780.739	699.365	5	MLPPLPDFSLSVEQQFDLQK_heavy
780.739	786.424	15	MLPPLPDFSLSVEQQFDLQK_heavy
780.739	915.461	6	MLPPLPDFSLSVEQQFDLQK_heavy
780.739	1048.543	13	MLPPLPDFSLSVEQQFDLQK_heavy
780.739	1142.593	20	MLPPLPDFSLSVEQQFDLQK_heavy
780.739	1429.741	25	MLPPLPDFSLSVEQQFDLQK_heavy

Table S1. Transitions used for targeted SRM quantitation of NblA.

	Protein	protein mass	modification mass	putative identification of modification
ApcA M744_01380	2-161:full length (Met excision)	17279.9	0.0	unmodified
		17418.0	138.1	degraded PCB (α)
		17434.0	154.1	degraded PCB (α) + O
		17596.1	316.1	degraded PCB (αβ)
		17611.1	331.2	degraded PCB (αβ) +O
		17758.2	478.2	degraded PCB (αβγ)
		17775.2	495.2	degraded PCB (αβγ) + O
		17792.2	512.3	degraded PCB (αβγ) + 2O
		17865.2	585.3	PCB (αβγδ)
		17881.2	601.3	PCB (αβγδ) + O
		17897.2	617.3	PCB (αβγδ) + 2O
		17914.2	634.3	PCB (αβγδ) + 3O
		18049.2	769.2	degraded PCB (αβγ) +GSH -me
		18170.3	890.3	PCB (αβγδ) + GSH
		18203.3	923.4	PCB (αβγδ) + GSH +2O
ApcB M744_01385	1-161:full length	17534.0	152.1	degraded PCB (α) + me
		17550.0	168.1	degraded PCB (α) + me + O
		17698.1	316.2	degraded PCB (αβ)
		17875.2	493.2	degraded PCB (αβγ) + me
		17981.2	599.3	PCB (αβγδ) + me
		17997.2	615.3	PCB (αβγδ) + me + O
		18013.2	631.4	PCB (αβγδ) + me + 2O
		18318.3	936.4	PCB (αβγδ) + me + 2O + GSH
ApcC M744_01390	1-67:full length	8109.2	305.0	GSH
ApcD M744_01830	2-163:full length (Met excision)	18574.0	585.6	PCB (αβγδ)
ApcF M744_05565	1-169:full length	19159.9	599.4	PCB (αβγδ) + me
CpcA M744_11415	2-163:full length (Met excision)	17284.8	138.1	degraded PCB (α)
		17462.8	316.2	degraded PCB (αβ)
		17624.9	478.2	degraded PCB (αβγ)
		17731.9	585.2	PCB (αβγδ)
		17763.9	617.3	PCB (αβγδ) + O
	29-163:truncation	14526.3	138.1	degraded PCB (α)
		14704.4	316.2	degraded PCB (αβ)
		14866.5	478.2	degraded PCB (αβγ)
		14973.5	585.3	PCB (αβγδ)
		15005.5	617.3	PCB (αβγδ) + O
CpcB M744_11420 (two PCB sites)	98-120: truncation	2846.3	305.1	GSH
	96-120: truncation	3080.4	305.1	GSH
	2-31: truncation	3236.6	-18.0	water loss
	130-159: truncation	3242.7	139.1	degraded PCB (α)

	2-32: truncation	3335.7	-18.0	water loss
	2-33: truncation	3424.8	0.0	
		3477.7	52.9	possible Fe^{3+} on Q
	121-162: truncation	4293.2	139.1	degraded PCB (α)
	2-42: truncation	4437.3	0.0	
	12-54: truncation	4524.3	-17.0	Pyro-glutamic acid
		4541.4	0.0	
	2-43: truncation	4551.3	0.0	
	2-47: truncation	4978.6	0.0	
	2-48: truncation	5092.6	0.0	
	121-173: truncation	5387.8	139.1	degraded PCB (α)
	2-54: truncation	5620.9	0.0	
	124-173: truncation	5727.9	748.4	unknown
		5850.0	870.4	unknown
	2-73: truncation	7494.9	-4.0	unknown near C-term
		7512.9	14.0	methylation
	2-120: truncation	13495.9	599.3	PCB (αβγδ) + me
	2-173: full length	19312.9	1185.6	PCB (αβγδ)*2 + me
		19619.0	1491.7	PCB (αβγδ)*2 + me + GSH
CpcD M744_11425	29-53: truncation	2896.5	0.0	
	18-43: truncation	3134.6	0.0	
	17-43: truncation	3221.6	0.0	
	54-81: truncation	3249.8	0.0	
	17-44: truncation	3278.6	0.0	
	17-47: truncation	3606.8	0.0	
	17-48: truncation	3753.9	0.0	
	10-43: truncation	3833.9	0.0	
	9-43: truncation	3890.9	0.0	
	17-51: truncation	4078.1	0.0	
	17-52: truncation	4241.1	0.0	
	17-53: truncation	4312.2	0.0	
	17-54: truncation	4468.3	0.0	
	1-43: truncation	4690.4	0.0	
		4706.4	16.0	+O
	17-58: truncation	4971.4	0.0	
	1-47: truncation	5075.6	0.0	
	1-48: truncation	5222.6	0.0	
	17-60: truncation	5240.6	0.0	
	25-71: truncation	5496.9	0.0	
	23-71: truncation	5725.0	0.0	
	22-71: truncation	5888.0	0.0	
	17-66: truncation	5950.1	0.0	

		5966.1	16.0	+O
	17-67: truncation	6021.1	0.0	
		6037.1	16.0	+O
	17-70: truncation	6389.4	0.0	
	17-71: truncation	6476.4	0.0	
		6492.4	16.0	+O
	22-81: truncation	6955.6	0.0	
	10-71: truncation	7088.7	0.0	
	9-71: truncation	7145.7	0.0	
	1-66: truncation	7418.8	0.0	
	1-67: truncation	7489.9	0.0	
	17-81: truncation	7543.9	0.0	
		7559.9	16.0	+O
		7596.8	52.9	Fe^{3+} (52.92) near C-terminus
	3-71: truncation	7701.0	0.0	
	1-70: truncation	7858.1	0.0	
	1-71: truncation	7945.2	0.0	
		7961.2	16.0	+O
		7977.2	32.0	+2O
		7998.1	52.9	Fe^{3+} (52.92)
	10-81: truncation	8156.3	0.0	
	9-81: truncation	8213.3	0.0	
	1-79: truncation	8750.6	-18.0	water loss
	3-81: truncation	8768.6	0.0	
	1-81: full length	8879.7	-18.0	water loss
		9012.7		
		9028.7	16.0	+O
		9044.7	32.0	+2O
		9065.6	52.9	Fe^{3+} (52.92) near C-terminus
		9111.6	98.9	Sulfate addition
NblA M744_05740	1-59: full length	7046.6		
	3-59: truncation	6802.5		

Table S2. Identified proteoforms of PBS proteins with top-down proteomics workflow (PCB – phycocyanobilin pigment; O – oxdiation; GSH – glutathione; me – methylation). The partially degraded PCBs are annotated with Greek alphabets as discussed in Figure 3.

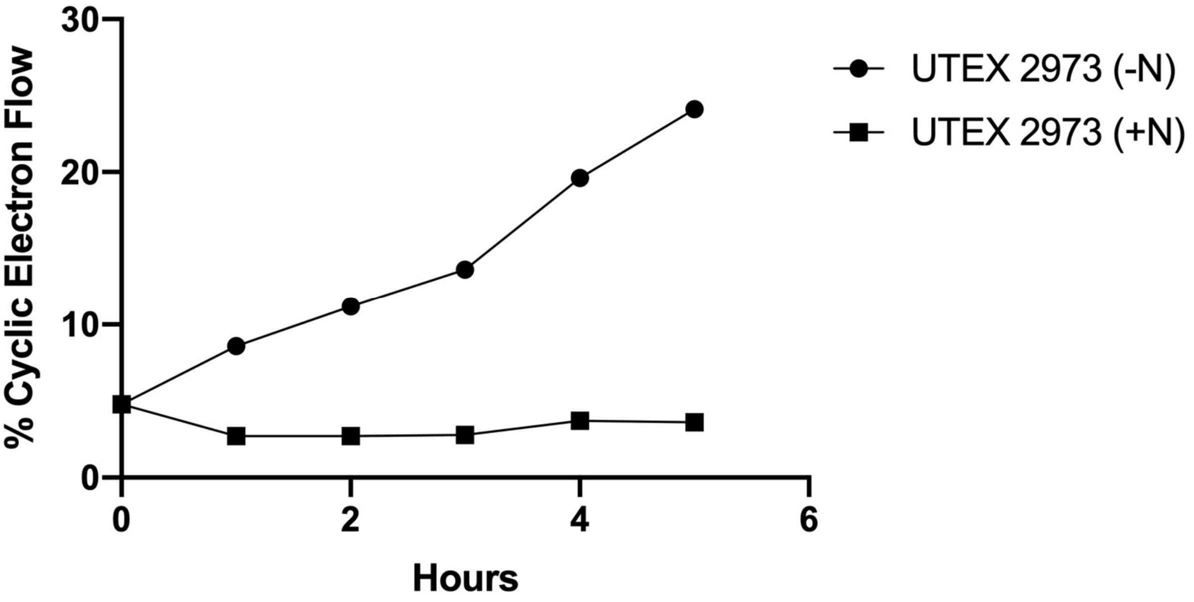

Figure S1. Cyclic electron flow in UTEX 2973 under nitrogen depletion (-N) and repletion (+N).

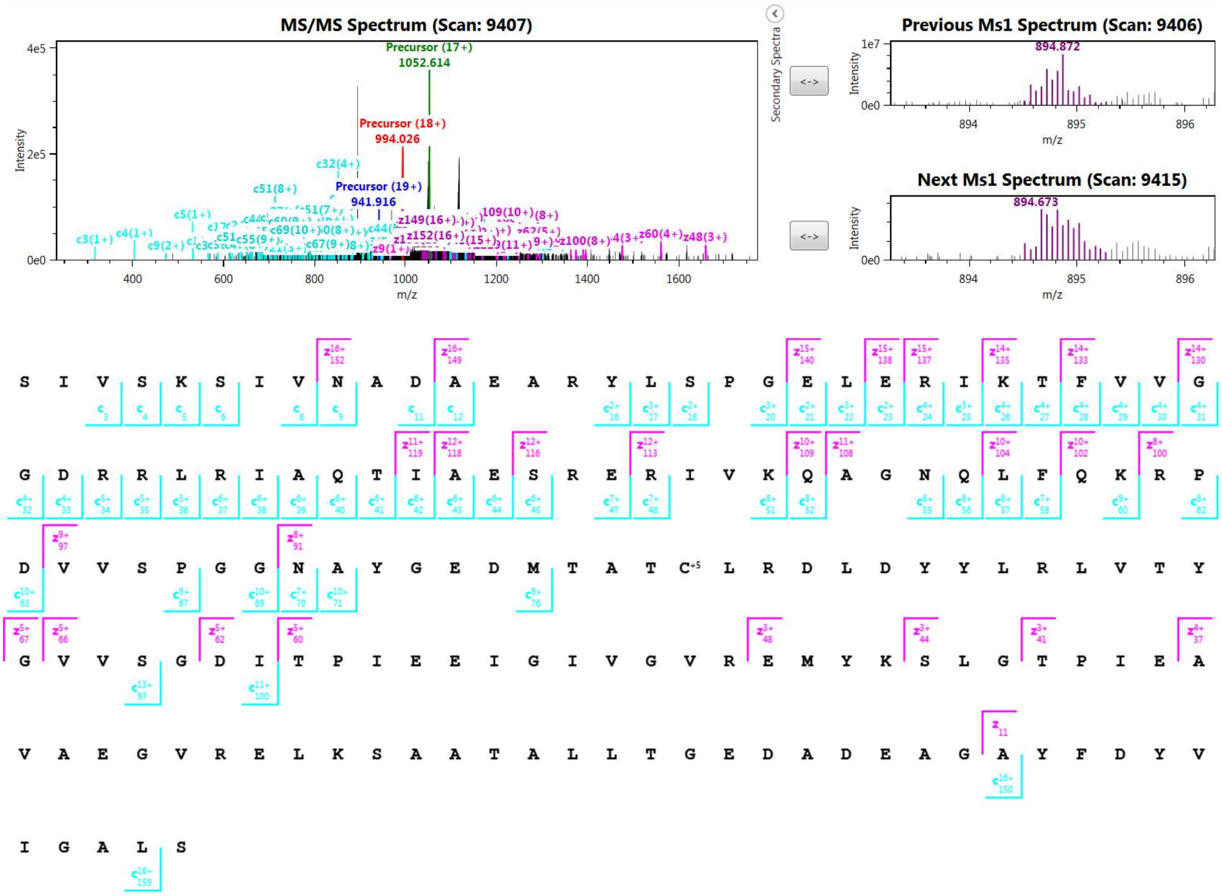

Figure S2. Example data for identified ApcA proteoform with one PCB on Cys81 (File: UTEX2973_topD_09_lumos_29Sep16_Bane_16-04-17, scan 9407, charge state +20). Top panel showed the annotated MS/MS spectrum on the left, and the adjacent MS spectra zoomed to the precursor regions. Bottom panel showed the coverage map.

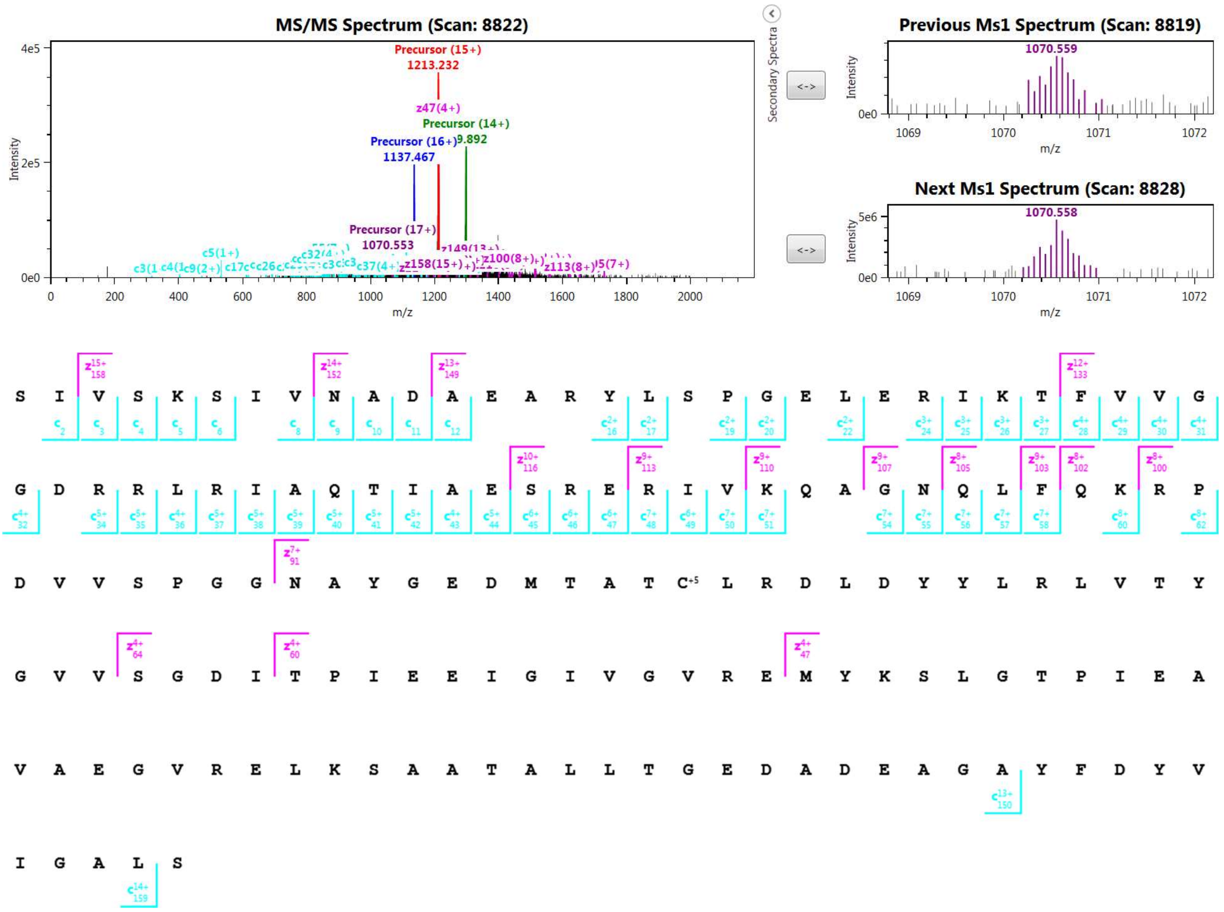

Figure S3. Example data for identified ApcA proteoform with modification mass of 890Da, which is tentatively assigned to one PCB and one GSH on Cys81 (File: UTEX2973_topD_13_lumos_29Sep16_Bane_16-04-17, scan 8822, charge state +17). PCB and GSH are both known to only be linked to Cys. Because there is only one Cys in ApcA, we suspect the GSH maybe added on to the PCB, or GSH may be linked to other residues nearby, both via unknown chemistry. Top panel showed the annotated MS/MS spectrum on the left, and the adjacent MS spectra zoomed to the precursor regions. Bottom panel showed the coverage map.

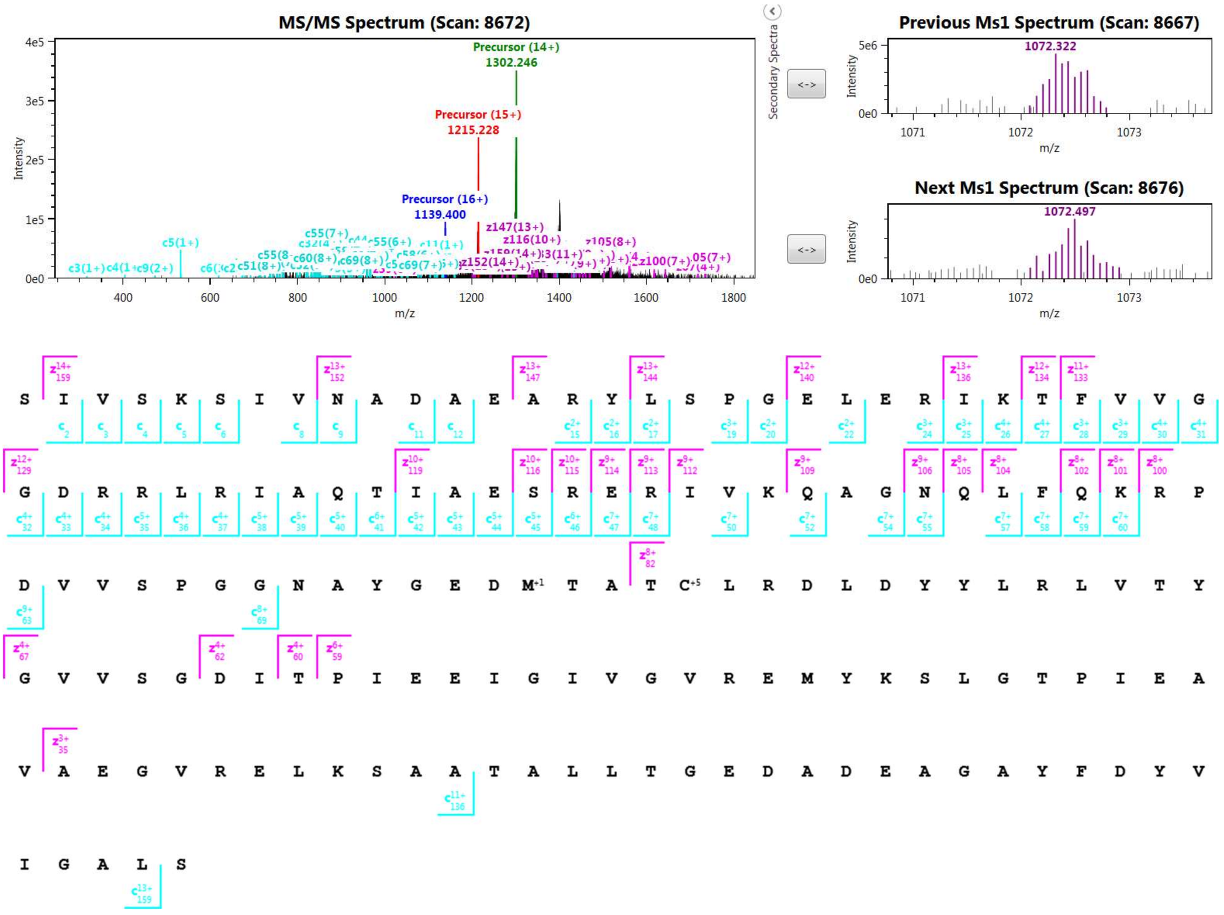

Figure S4. Example data for identified ApcA proteoform with modification mass of 923Da, which is tentatively assigned to one PCB and one GSH on Cys81, and two oxidations on Met77 (File: UTEX2973_topD_21_lumos_29Sep16_Bane_16-04-17, scan 8671, charge state +17). Top panel showed the annotated MS/MS spectrum on the left, and the adjacent MS spectra zoomed to the precursor regions. Bottom panel showed the coverage map.

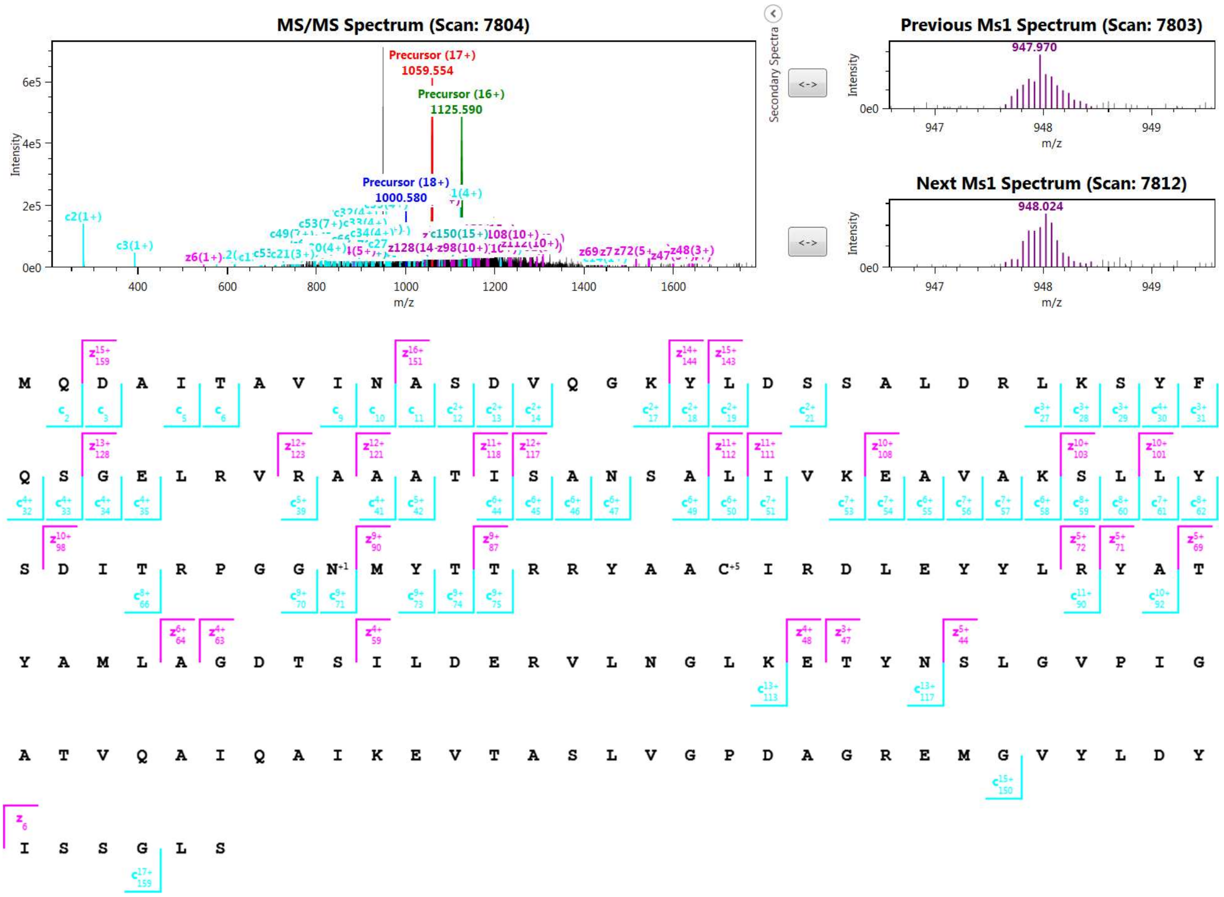

Figure S5. Example data for identified ApcB proteoform with one PCB on Cys81, and one methyl on N71 (File: UTEX2973_topD_21_lumos_29Sep16_Bane_16-04-17, scan 7404, charge state +19). Top panel showed the annotated MS/MS spectrum on the left, and the adjacent MS spectra zoomed to the precursor regions. Bottom panel showed the coverage map.

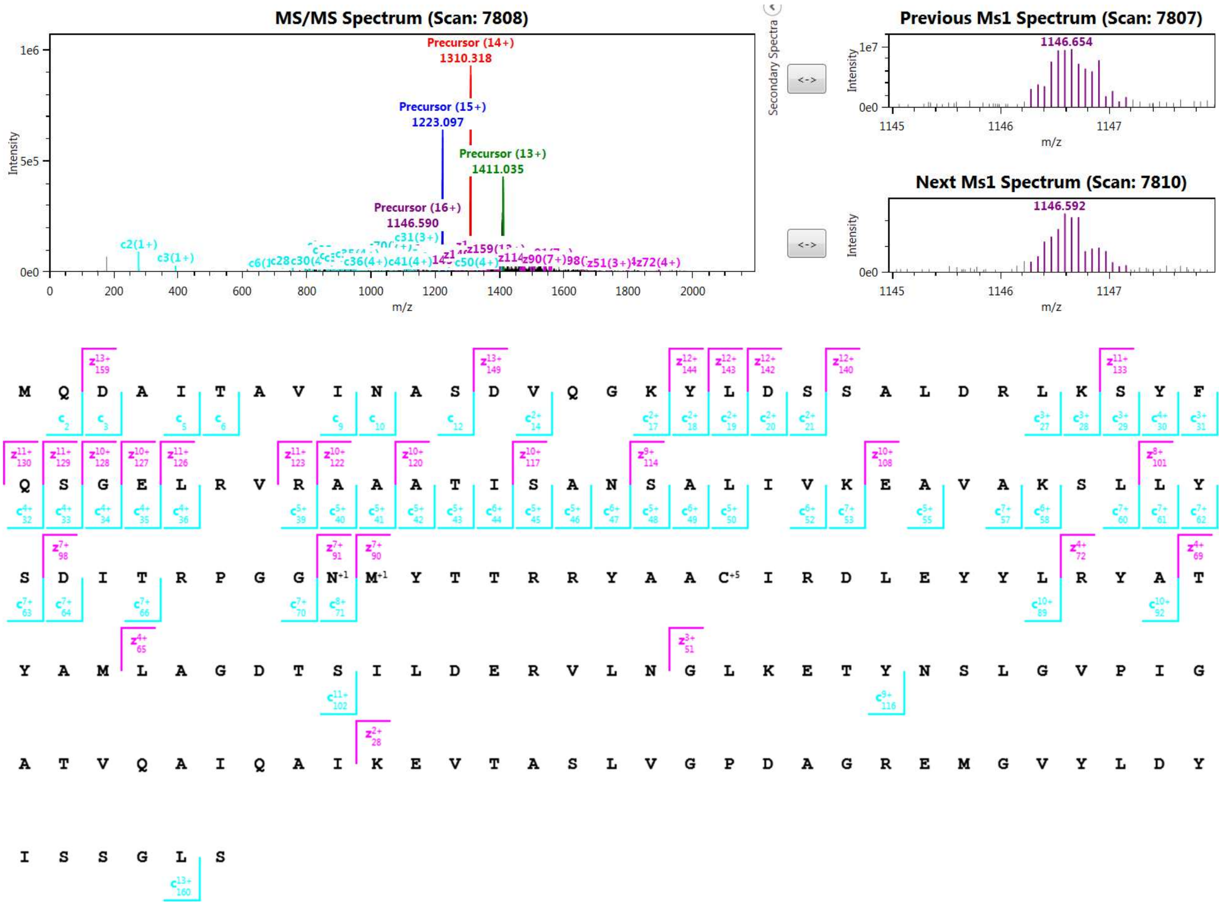

Figure S6. Example data for identified ApcB proteoform with modification mass of 936Da, which is tentatively assigned to one PCB and one GSH on Cys81, one methyl on Asn71, and two oxidation on Met72, (File: UTEX2973_topD_25_lumos_29Sep16_Bane_16-04-17, scan 7808, charge state +16). One of the two oxidations could potentially be assigned to Met96 without significantly reducing the sequence coverage. Similar to the ApcA species assigned to carry modifications with both GSH and PCB, the chemistry forming this ApcB specis is unclear because only one Cys is in the sequence. Top panel showed the annotated MS/MS spectrum on the left, and the adjacent MS spectra zoomed to the precursor regions. Bottom panel showed the coverage map.

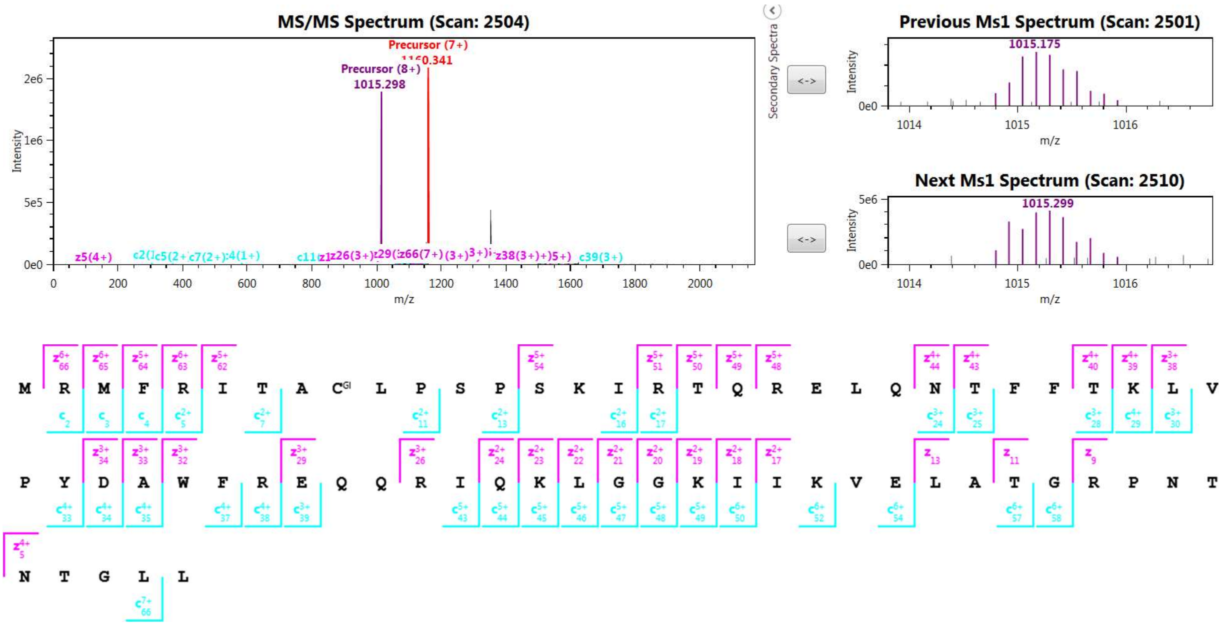

Figure S7. Example data for identified ApcC proteoform with glutathione on Cys9 (File: UTEX2973_topD_25_lumos_29Sep16_Bane_16-04-17, scan 2504, charge state +8). All detected, high confidence proteoforms of ApcC had the glutathione modification. Top panel showed the annotated MS/MS spectrum on the left, and the adjacent MS spectra zoomed to the precursor regions. Bottom panel showed the coverage map.

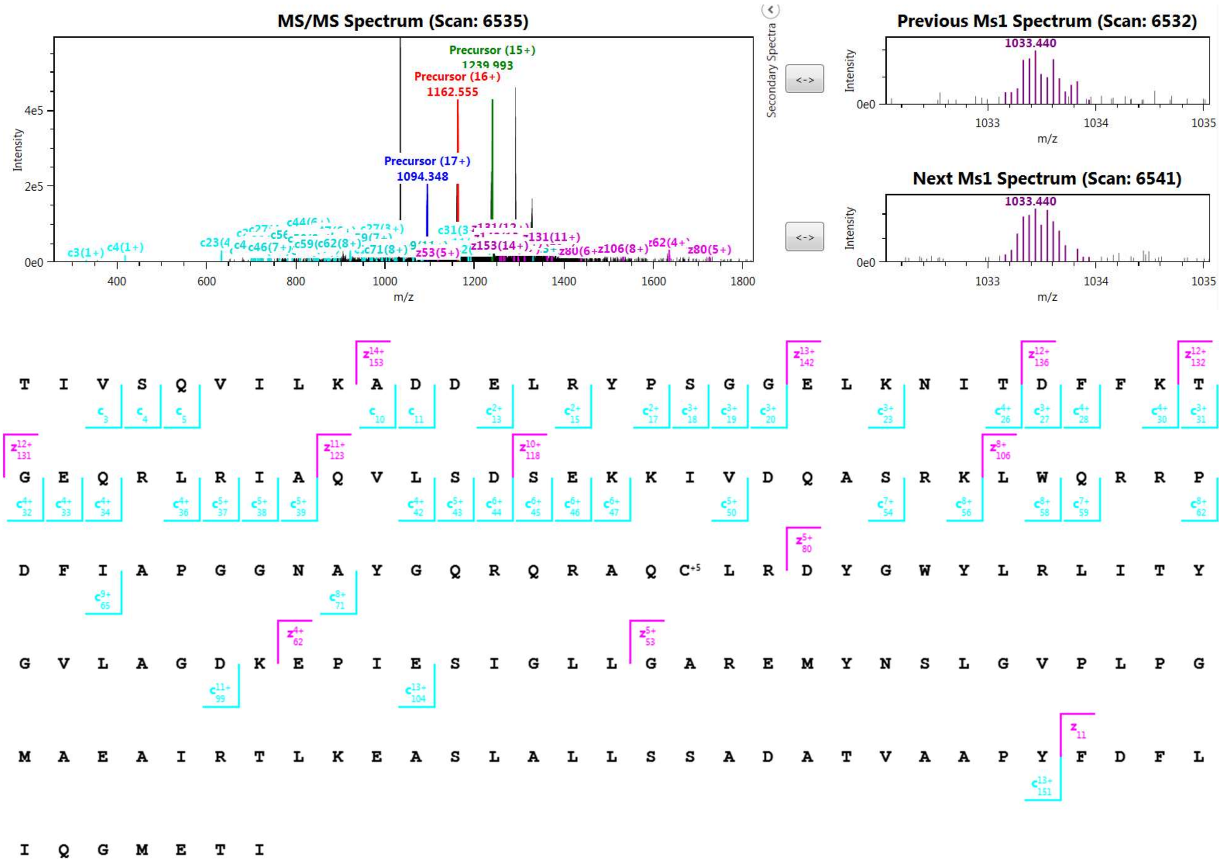

Figure S8. Example data for identified ApcD proteoform with one PCB on Cys81 (File: UTEX2973_topD_21_lumos_29Sep16_Bane_16-04-17, scan 6535, charge state +18). Top panel showed the annotated MS/MS spectrum on the left, and the adjacent MS spectra zoomed to the precursor regions. Bottom panel showed the coverage map.

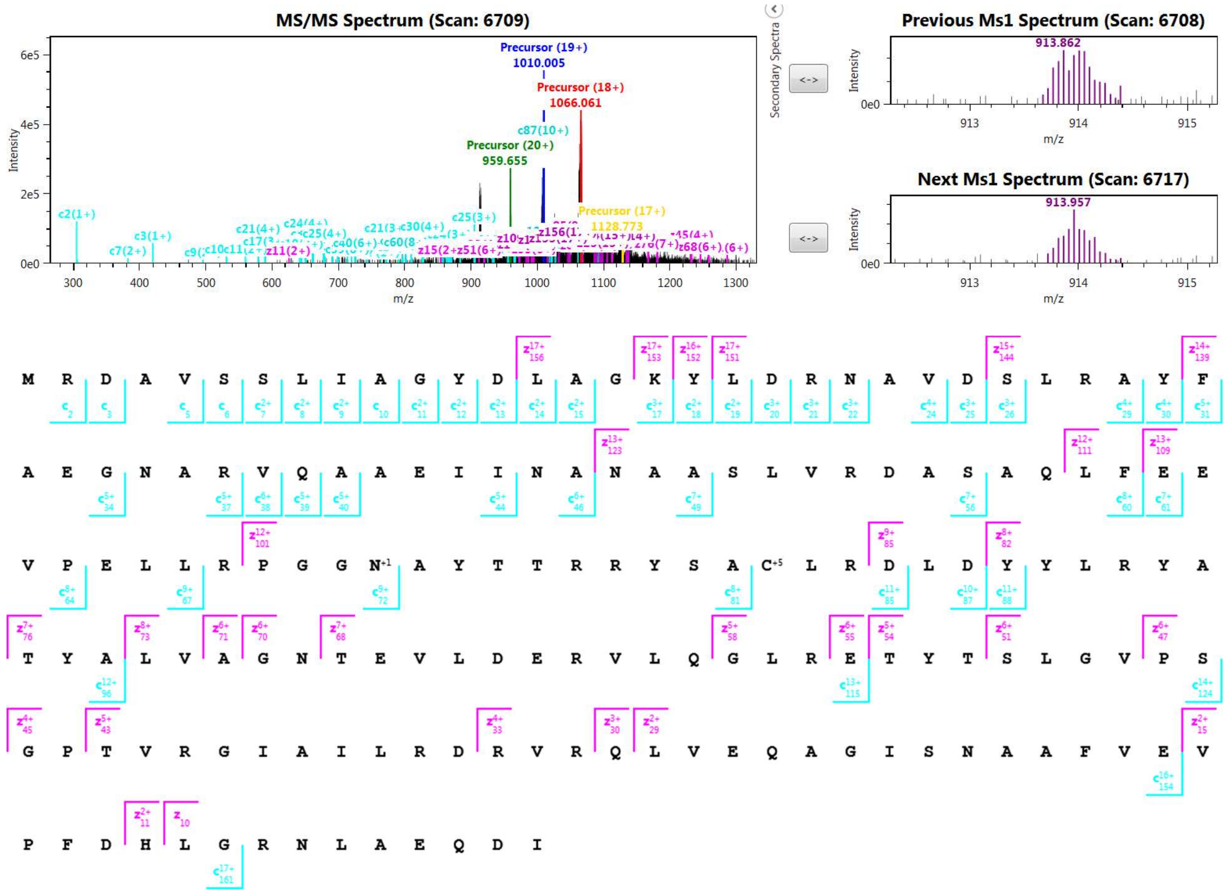

Figure S9. Example data for identified ApcF proteoform with one PCB on Cys82, and one methyl on N72 (File: UTEX2973_topD_21_lumos_29Sep16_Bane_16-04-17, scan 6709, charge state +21). Top panel showed the annotated MS/MS spectrum on the left, and the adjacent MS spectra zoomed to the precursor regions. Bottom panel showed the coverage map.

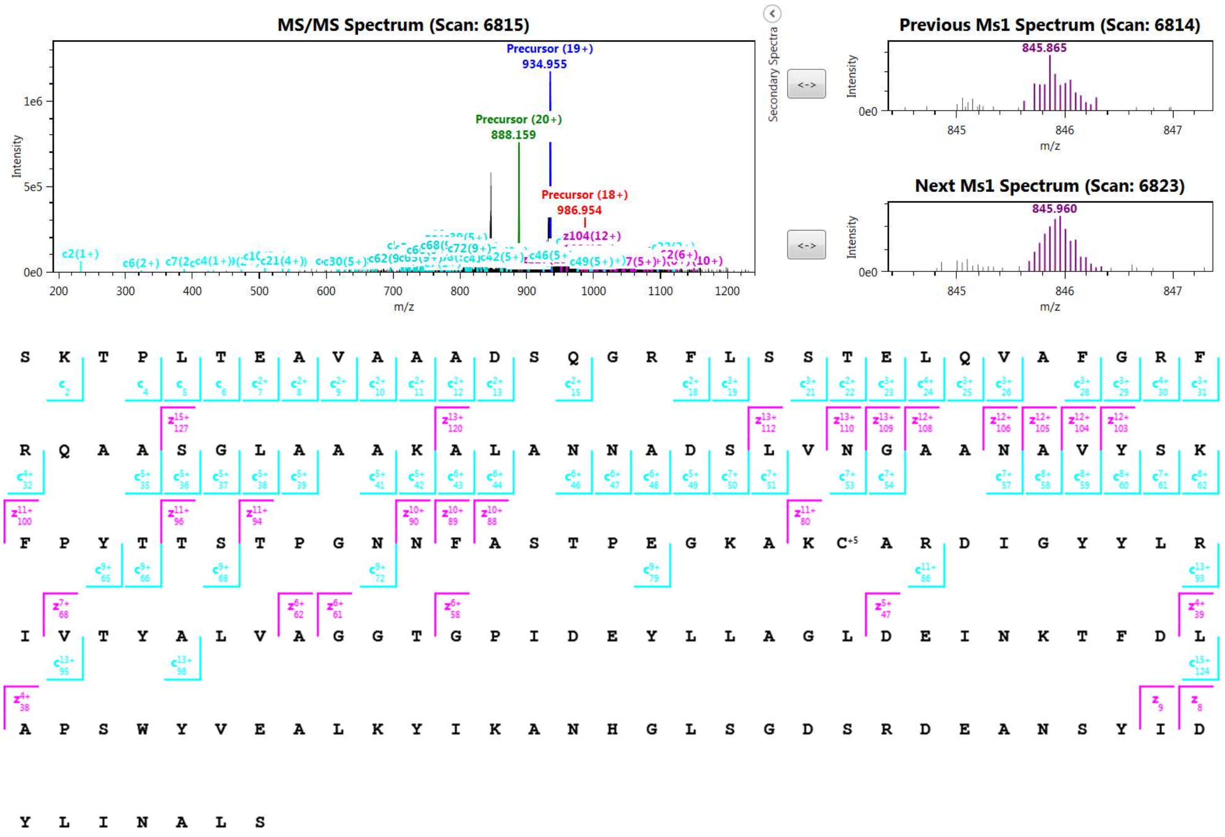

Figure S10. Example data for identified CpcA proteoform with one PCB on Cys85 (File: UTEX2973_topD_14_lumos_29Sep16_Bane_16-04-17, scan 6815, charge state +21). Top panel showed the annotated MS/MS spectrum on the left, and the adjacent MS spectra zoomed to the precursor regions. Bottom panel showed the coverage map.

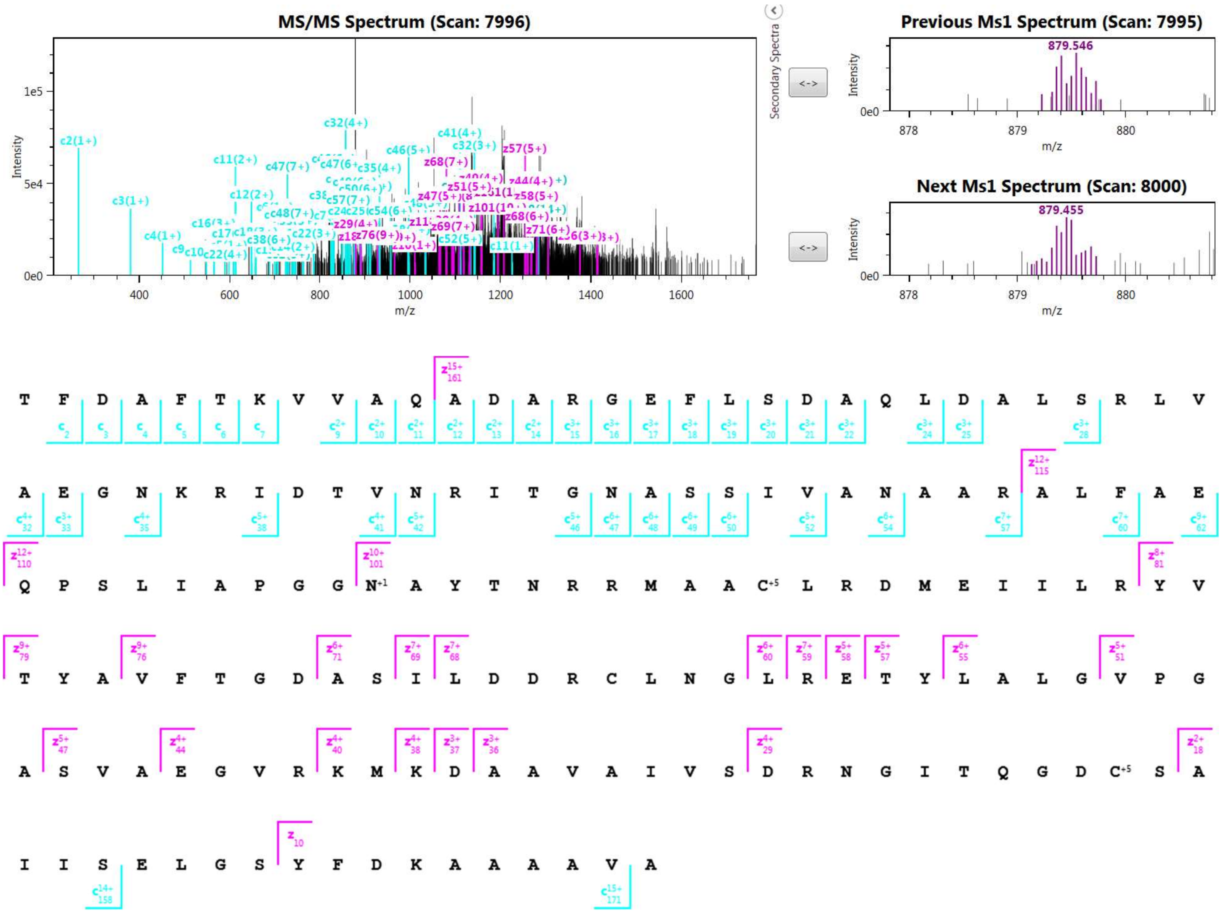

Figure S11. Example data for identified CpcB proteoform with methyl on N73, two PCBs on Cys83 and Cys154 (File: UTEX2973_topD_19_lumos_29Sep16_Bane_16-04-17, scan 7996, charge state +22). Modification sites were assigned based on the coverage and the preknowledge of the modifications. Top panel showed the annotated MS/MS spectrum on the left, and the adjacent MS spectra zoomed to the precursor regions. Bottom panel showed the coverage map.

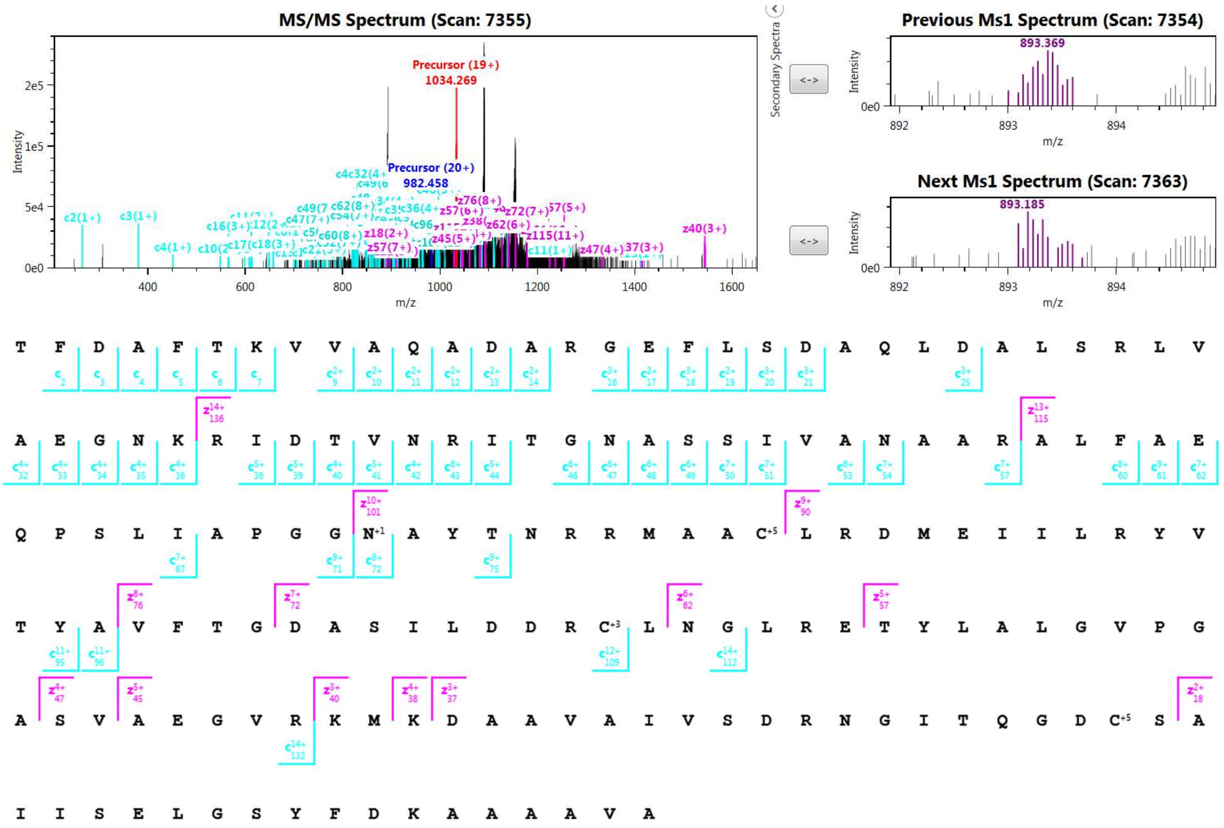

Figure S12. Example data for identified CpcB proteoform with methyl on N73, two PCBs on Cys83 and Cys154, and glutathione on Cys110 (File: UTEX2973_topD_18_lumos_29Sep16_Bane_16-04-17, scan 7355, charge state +22). Although the coverage is not sufficient to confidently assign all modifications sites, we did detect CpcB fragments with PCB on Cys154, methyl on N73, and glutathione on Cys110. Data on these degraded CpcB fragments supported the assignment of modifications on the full-length CpcB. Top panel showed the annotated MS/MS spectrum on the left, and the adjacent MS spectra zoomed to the precursor regions. Bottom panel showed the coverage map.

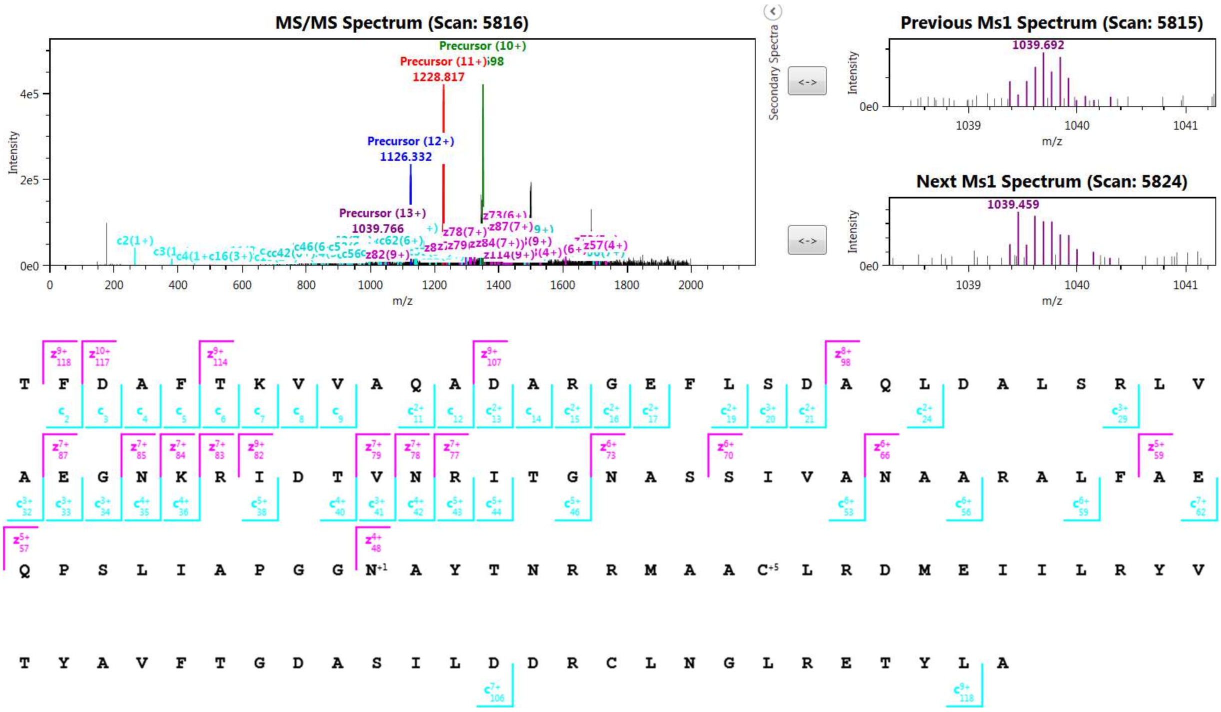

Figure S13. Example data for the truncated CpcB proteoform (residue 2-120) with methyl on N73, one PCs on Cys83 (File: UTEX2973_topD_05_lumos_29Sep16_Bane_16-04-17, scan 5816, charge state +13). The sites of modifications were assigned based on the knowledge of the protein due to the limited coverage near the C-terminus of the proteins. Top panel showed the annotated MS/MS spectrum on the left, and the adjacent MS spectra zoomed to the precursor regions. Bottom panel showed the coverage map.

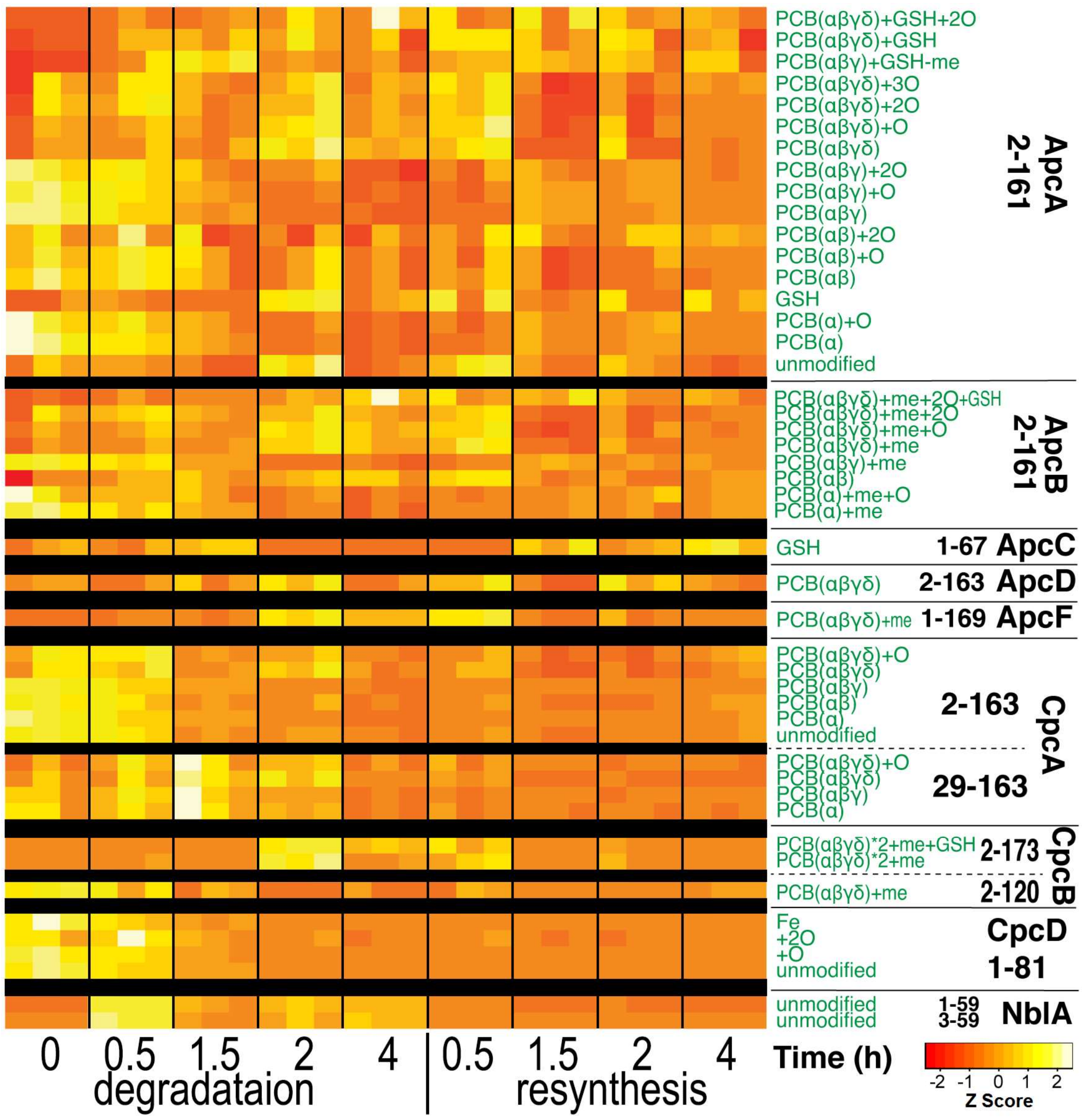

Figure S14. Heatmap for abundance changes of proteoforms including all replicates. Three replicates for each time points are grouped and separated from other time points by vertical black lines. In general the replicates are consistent with each other.

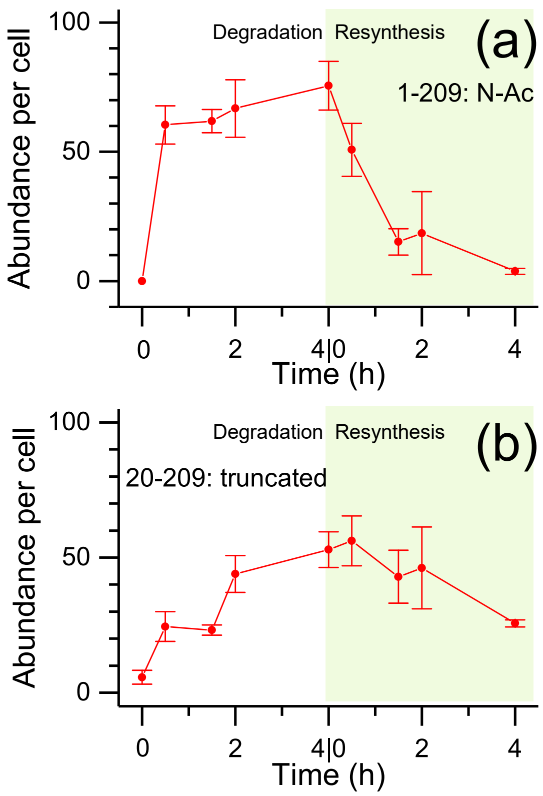

Figure S15. Abundance change across time points for M744_01170 chemotaxis protein CheY (a) full length with N-terminal acetylation, and (b) N-terminally truncated form (residue 20-209).

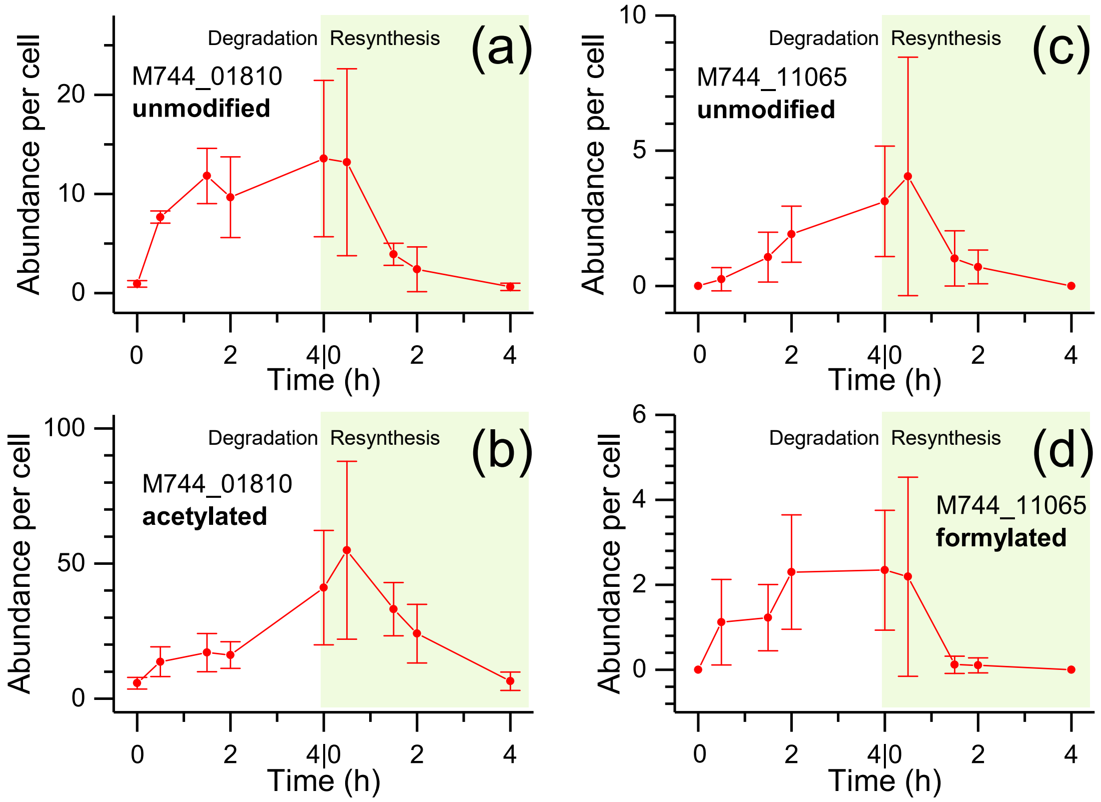

Figure S16. Abundance change across time points for two highlight inducible proteins (a-b) M744_01810, and (c-d) M744_11065.

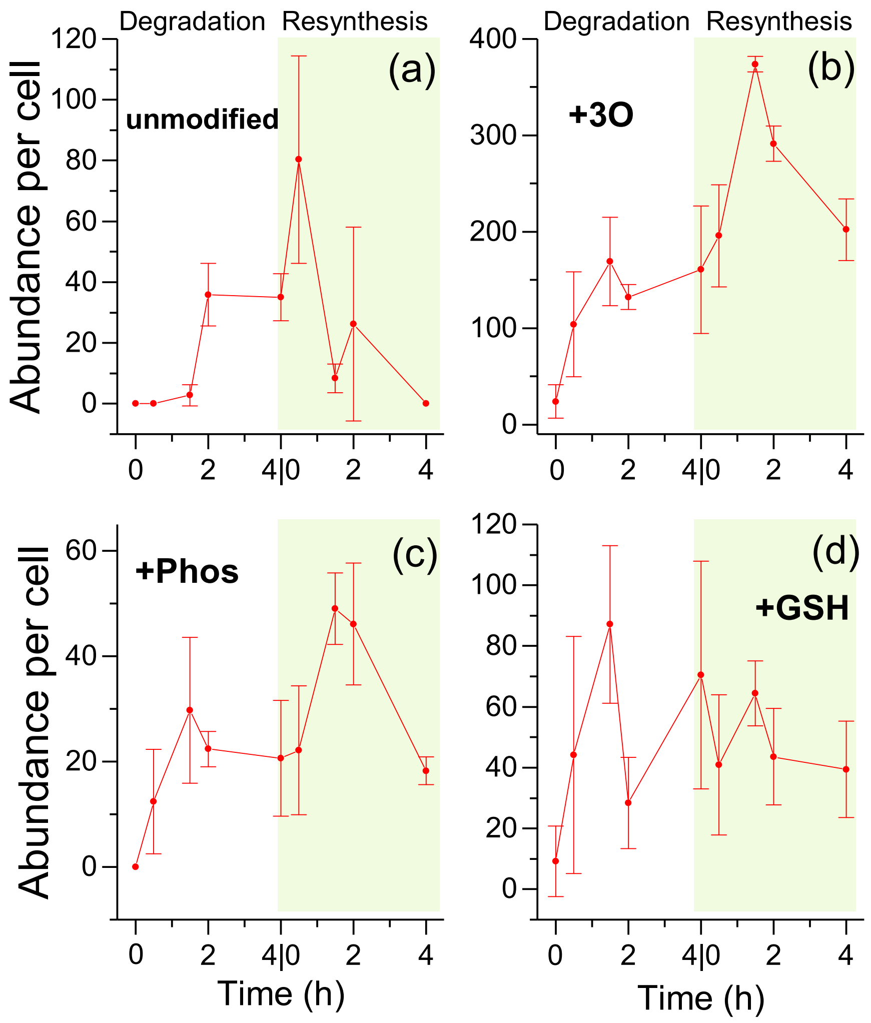

Figure S17. Abundance change across time points for the major proteoforms of M744_12535. This protein blasted to nitrogen starvation response protein.

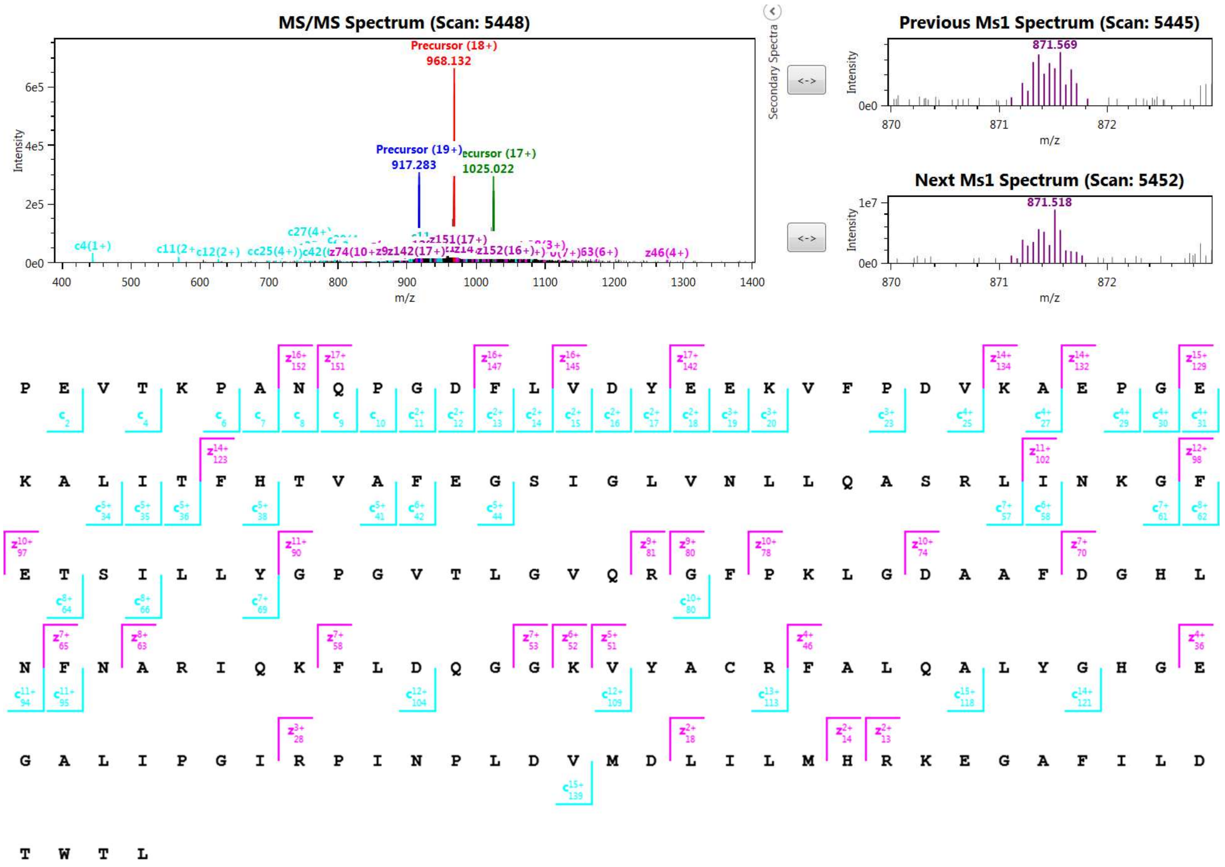

Figure S18. Example data for the nitrogen starvation response protein M744_12535 without modification (File: UTEX2973_topD_29_lumos_29Sep16_Bane_16-04-17, scan 5448, charge state +20). Top panel showed the annotated MS/MS spectrum on the left, and the adjacent MS spectra zoomed to the precursor regions. Bottom panel showed the coverage map.

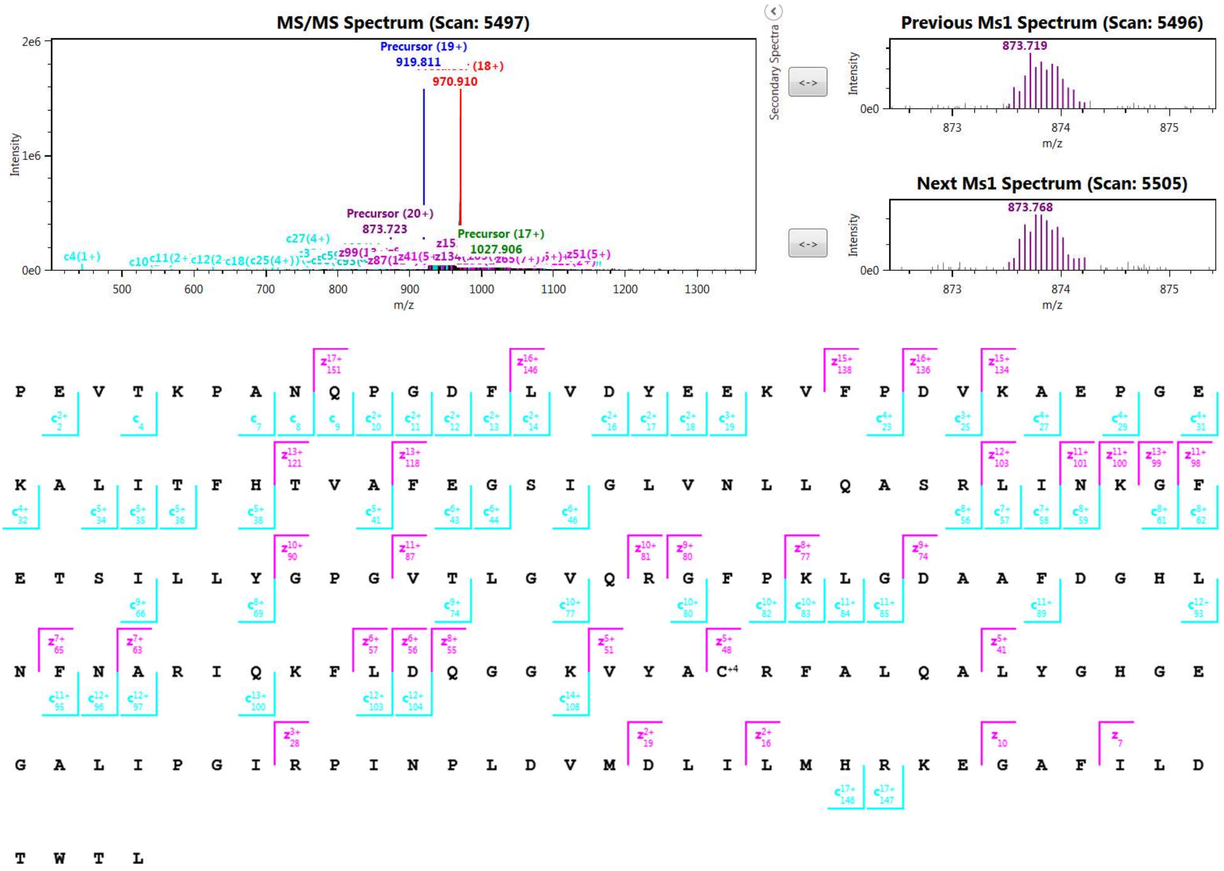

Figure S19. Example data for the nitrogen starvation response protein M744_12535 with +48.0 Da (File: UTEX2973_topD_25_lumos_29Sep16_Bane_16-04-17, scan 5497, charge state +20), which is assigned as trioxidation on Cys113. Top panel showed the annotated MS/MS spectrum on the left, and the adjacent MS spectra zoomed to the precursor regions. Bottom panel showed the coverage map.

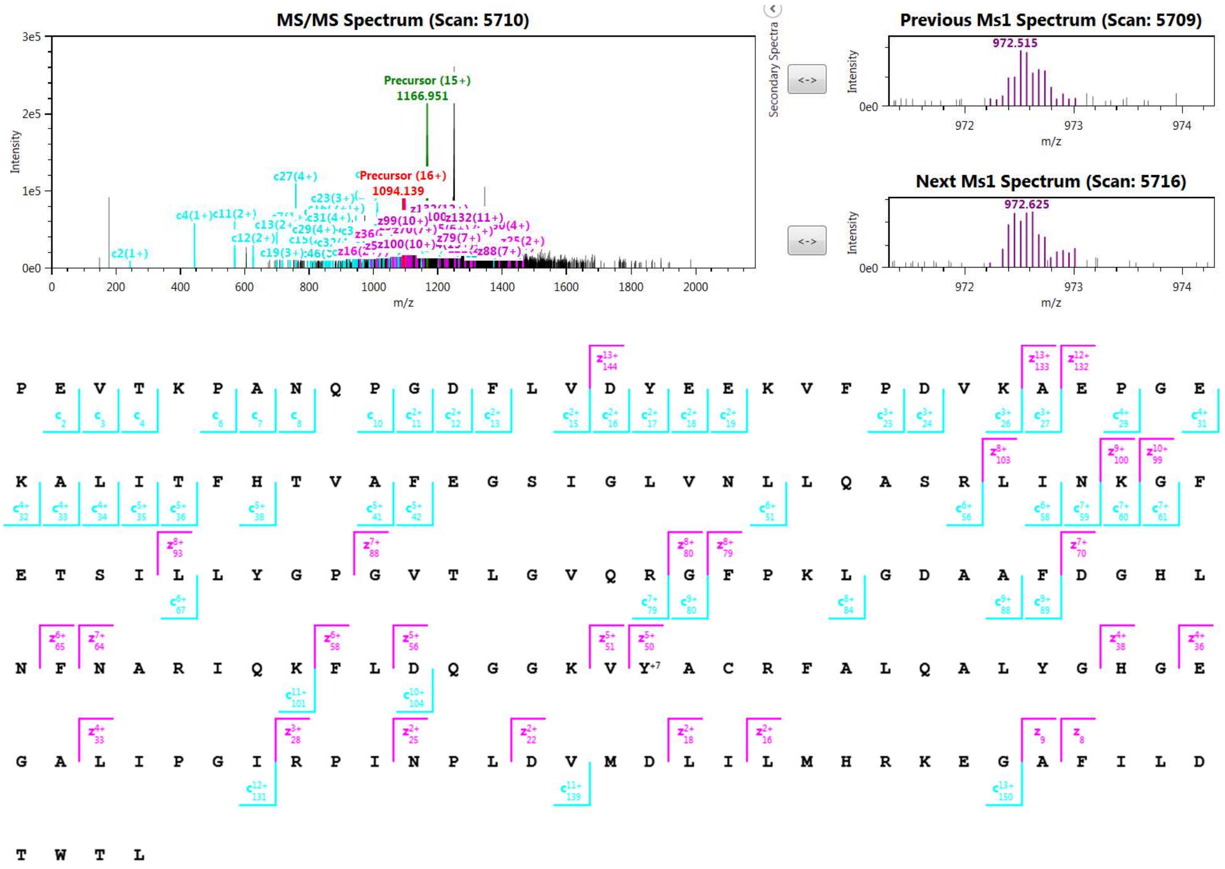

Figure S20. Example data for the nitrogen starvation response protein M744_12535 with 79.96 Da modification on Tyr111 (File: UTEX2973_topD_26_lumos_29Sep16_Bane_16-04-17, scan 5710, charge state +18). The PTM is likely to be phosphorylation (+79.966), but it may be assigned as sulphation (+79.957 Da) as well. Top panel showed the annotated MS/MS spectrum on the left, and the adjacent MS spectra zoomed to the precursor regions. Bottom panel showed the coverage map.

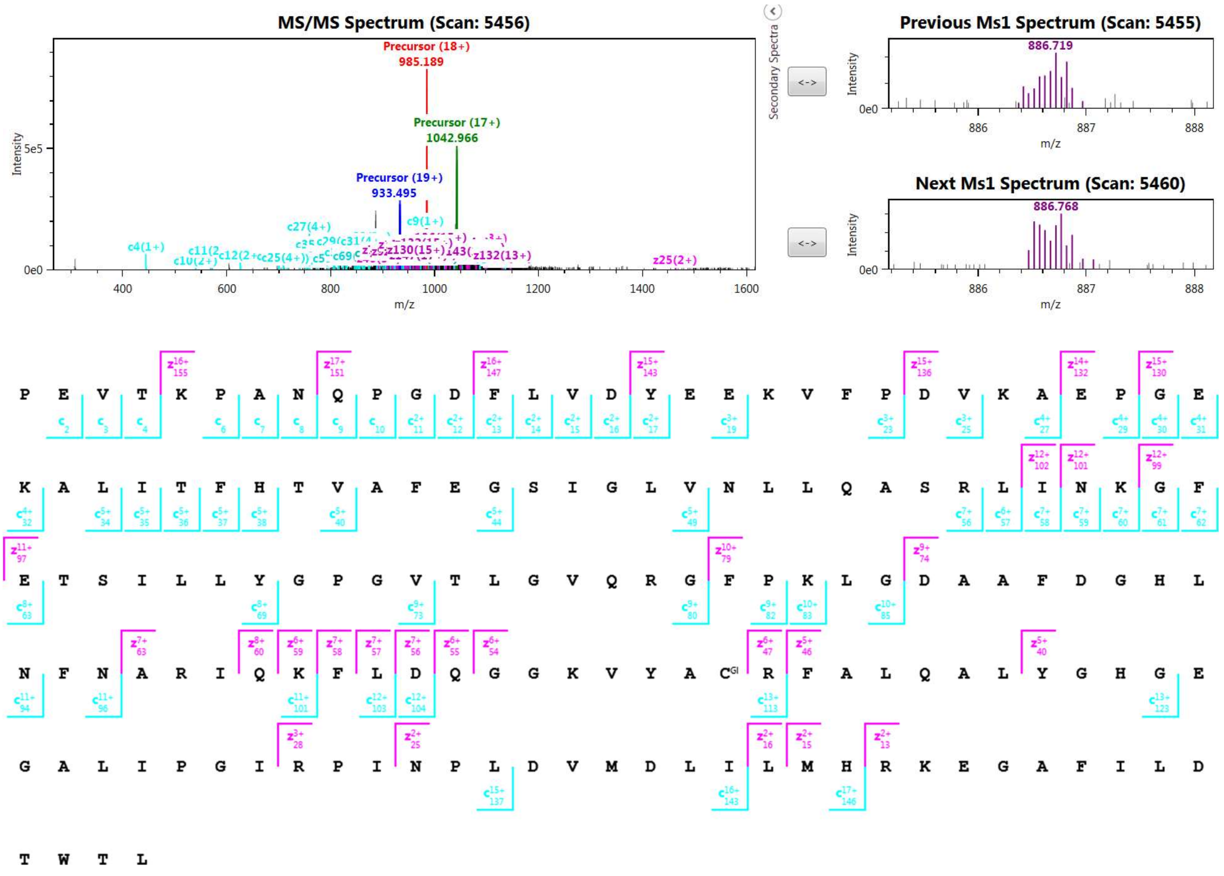

Figure S21. Example data for the identified nitrogen starvation response protein M744_12535 with glutathionation on Cys113 (File: UTEX2973_topD_17_lumos_29Sep16_Bane_16-04-17, scan 5456, charge state +20). Top panel showed the annotated MS/MS spectrum on the left, and the adjacent MS spectra zoomed to the precursor regions. Bottom panel showed the coverage map.
